# Cold stress and freezing tolerance negatively affect the fitness of *Arabidopsis thaliana* accessions under field and controlled conditions

**DOI:** 10.1007/s00425-021-03809-8

**Published:** 2022-01-15

**Authors:** Maximilian Boinot, Esra Karakas, Karin Koehl, Majken Pagter, Ellen Zuther

**Affiliations:** 1 Max Planck Institute of Molecular Plant Physiology, Am Muehlenberg 1, 14476 Potsdam, Germany; 2 Department of Chemistry and Bioscience, Aalborg University, 9220 Aalborg East, Denmark

**Keywords:** Cold tolerance, Field experiments, Natural variation, Overwintering, Priming, Seed yield

## Abstract

**Main conclusion:**

Higher acclimated freezing tolerance improved winter survival, but reduced reproductive fitness of *Arabidopsis thaliana* accessions under field and controlled conditions.

**Abstract:**

Low temperature is one of the most important abiotic factors influencing plant fitness and geographical distribution. In addition, cold stress is known to influence crop yield and is therefore of great economic importance. Increased freezing tolerance can be acquired by the process of cold acclimation, but this may be associated with a fitness cost. To assess the influence of cold stress on the fitness of plants, long-term field trials over 5 years were performed with six natural accessions of *Arabidopsis thaliana* ranging from very tolerant to very sensitive to freezing. Fitness parameters, as seed yield and 1000 seed mass, were measured and correlation analyses with temperature and freezing tolerance data performed. The results were compared with fitness parameters from controlled chamber experiments over 3 years with application of cold priming and triggering conditions. Winter survival and seed yield per plant were positively correlated with temperature in field experiments. In addition, winter survival and 1000 seed mass were correlated with the cold-acclimated freezing tolerance of the selected Arabidopsis accessions. The results provide strong evidence for a trade-off between higher freezing tolerance and reproductive fitness in *A. thaliana*, which might have ecological impacts in the context of global warming.

**Supplementary Information:**

The online version contains supplementary material available at 10.1007/s00425-021-03809-8.

## Introduction

Temperature is among the most important abiotic factors that influence the geographical distribution, growth and fitness of plants. High as well as low temperatures decrease the yield of annual crops (Yadav 2010; Sanghera et al. 2011; Powell et al. 2012) and delimit their natural occurrence (Kreyling et al. 2015). In addition to agricultural productivity, changing temperatures as consequence of proceeding climate change affect biodiversity and ecosystems dynamics (Anderson et al. 2012).

Plants have evolved mechanisms to cope with cold stress. In late autumn and the beginning of winter, when air temperatures decrease, plants increase their freezing tolerance after exposure to low, but non-freezing temperatures in a seasonal process called cold acclimation (Levitt 1980). Comprehensive energy-demanding and potentially costly metabolic and transcriptomic reprogramming accompanies this acclimation (Cook et al. 2004; Hannah et al. 2005; Maruyama et al. 2009; Hincha et al. 2012), which is partly driven by the CBF signal transduction pathway (for reviews, see Thomashow 2010; Liu et al. 2019). At the end of winter and beginning of spring, freezing tolerance of plants decreases in a process called deacclimation (Xin and Browse 2000). Balancing the deacclimation rate in spring with the initiation of growth and development is of high importance for both life-securing parameters, survival and fitness (Zuther et al. 2015), as these processes compete for resources, especially from carbon metabolism (Wingler 2014).

It was shown previously that plants memorize previous stresses, resulting in an improved response to recurring stress events, which may happen faster or earlier and be more sensitive or stronger than the first response (Hilker et al. 2016). The ability to develop a costly primed stress response often results in an individual fitness improvement, minimizing the costs of adjustments to future stress events (Hilker et al. 2016). For recurring cold stress events, an improved freezing tolerance was shown after a second cold stress event (trigger) for two different Arabidopsis accessions cold primed before under controlled conditions (Zuther et al. 2019).

Global climate change poses a great threat to plant fitness. Namely, the increase of mean surface air temperatures, variable winter climates and especially the occurrence of erratic temperature fluctuations in spring predicted by global climate models (IPCC 2014) augment the risks for plants to suffer from tissue damage and reduced yield through recurring stress events (Vyse et al. 2019). Thus, with progressively milder winters and irregular temperature patterns, the risk of a premature deacclimation and impaired winter survival rises (Pagter and Arora 2013; Kovi et al. 2016; Rapacz et al. 2017). This raises the question whether efficient cold priming and cold memory entail a fitness cost for the plants. A study monitoring 12 Arabidopsis accessions in controlled climate chamber experiments found no evidence for a higher fitness cost of tolerant compared to sensitive accessions due to cold priming (Zhen et al. 2011). In contrast, another study demonstrated high fitness costs of a freezing-tolerant accession when grown in a frost-free environment and for a freezing-sensitive accession under cold winter conditions in reciprocal transplant experiments with an Italian and a Swedish accession (Ågren and Schemske 2012). The costs of stress resistance might additionally depend on the environmental experimental conditions as also experiments without any fitness costs were reported (Heidel et al. 2004).

*Arabidopsis thaliana* (L.) is widely distributed in the Northern Hemisphere. Across this large range, the species displays great genetic variation, resulting in hundreds of different natural accessions that have been collected and described over the last century (Weigel 2012) making it a perfect model for ecological studies (Mitchell-Olds 2001). A wide range of freezing tolerance under non-acclimated and cold-acclimated conditions of plants grown under controlled conditions was described for a selection of 54 Arabidopsis accessions with different geographical origins (Zuther et al. 2012). In addition, specific deacclimation patterns were shown for 12 Arabidopsis accessions under controlled conditions (Zuther et al. 2015; Juszczak et al. 2016).

As plants in nature are typically exposed to a combination of environmental factors, field or common garden experiments are highly important for a real estimation of plant stress responses and fitness (Köhl and Laitinen 2015). Common garden experiments are frequently used to study environmental interactions with different genotypes (i.e. accessions), often on multiple sites (Rutter and Fenster 2007; Fournier-Level et al. 2011; Mishra et al. 2012; de Villemereuil et al. 2016). It was shown before that local adaptation and natural variation are influenced by temperature and precipitation gradients, and that genotype-by-environment interactions have an effect on fitness parameters (Fournier-Level et al. 2011; Hancock et al. 2011; Ågren and Schemske 2012; Exposito-Alonso et al. 2019). An understanding of parameters influencing the lifetime fitness will help to understand the ecological preferences and the response to environmental conditions of a certain species (Hu et al. 2017). Especially interesting are comparisons of plant fitness under field versus controlled chamber conditions (Malmberg et al. 2005; Mishra et al. 2012; Oakley et al. 2014).

The aim of this study was to assess the influence of cold temperature, especially of recurring cold stress events, on fitness parameters of natural accessions of *Arabidopsis thaliana*, ranging from very low to very high natural freezing tolerance. Multiyear studies under overwintering field (common garden) and controlled environmental conditions were performed with exposure to cold priming and/or triggering conditions for simulation of recurring cold stress events. Long-term phenotyping over 5 years was used for the detection of general fitness trends in response to changing winter climate. Fitness parameters of different genotypes were correlated with developmental parameters, weather data and freezing tolerance of cold-acclimated plants measured under controlled conditions (LT_50_ACC). Specifically, we asked the following questions: (1) Will lower temperatures generate higher fitness costs for the plants? (2) Is it costly for plants to develop an improved adaptation to cold and freezing temperatures? (3) Are these putative costs specific or the same under natural and controlled conditions? (4) Are there differences between different accessions regarding these questions?

## Materials and methods

### Plant cultivation

#### Field trials

For field experiments, 11 natural accessions of *A. thaliana* with different freezing tolerance (Zuther et al. 2012), ranging from very tolerant to very sensitive (Suppl. Table S1), were grown in 2013/14, 2014/15, 2015/16, 2017/18 and 2018/19. Seeds were originally obtained from Nottingham Arabidopsis Stock Center (NASC) (University of Nottingham, Loughborough, UK) and then further propagated at the MPI-MP. Plants were grown from seed batches propagated under uniform conditions either in 2013 (2013/14, 2014/15, 2015/16) or 2017 (2017/18, 2018/19) in polytunnels to secure the same parental seed environment.

A germination test was performed with seeds of the 2017 seed batch under similar germination conditions (Suppl. Table S2) and the appropriate number of seeds was counted at the Institute of Applied Genetics at the Free University of Berlin with an Elmor C3 seed-counting machine (Elmor AH, Schwyz, Switzerland). For field experiments, 100 seeds per accession were sown in October (Suppl. Table S3) in plastic boxes with holes (50 × 40 × 15 cm) (W × L × H) with sandy substrate “Haufen B” supplemented with 1 g Osmocote Start/1 L substrate that was sterilized prior to use. Five replicate boxes were prepared for each accession. Two weeks after sowing, boxes were transferred from the polytunnel to the field in Potsdam—Golm (52° 24′ N 13° 04′ E) following a random design and were aligned with a water spirit level to avoid uneven distribution of rain (Köhl and Laitinen 2015). Plants were grown under natural weather conditions except for some additional watering when necessary. In May of the following year, boxes were transferred back to the polytunnel and inflorescences bagged in groups for seed harvest. After 2 months, ripe seeds were collected and weighed for determination of total seed yield per plant.

### Greenhouse experiments

Five or six natural accessions of *A. thaliana* of the selection grown in field experiments were grown in controlled climate fitness experiments in 2015, 2018 and 2019. Plants were exposed to three different conditions: control (C), cold priming (P) and cold priming, lag phase and triggering (PT). In 2015 and 2018, 12 pots of 10 cm and, in 2019, 6 pots with three plants each were grown per accession and condition. Plants were sown and grown on soil in a climate chamber with 20 °C daytime temperature and 6 °C nighttime temperature in a 14 h light cycle with a light intensity of 180 μmol m^−2^ s^−1^ and a humidity of 60% at day and 70% at night. After 1 week, plants were moved to a short-day climate chamber with conditions as follows: 20 °C/16 °C day/night, 8 h day length, 180 μmol m^−2^ s^−1^, humidity of 60%/75% day/night. Plants were kept under these short-day conditions for a week before being pricked. After pricking, plants were kept for another 7 days under short-day conditions before being transferred to long-day conditions in a climate chamber, 20 °C/16 °C day/night, 16 h day length, 180 μmol m^−2^ s^−1^, humidity of 60%/75% day/night for 1 week. For cold priming (P), two-thirds of 21-day-old plants from each accession were placed in a cold chamber at 4 °C and a day length of 16 h with a light intensity of 90 μmol m^−2^ s^−1^ and a humidity of 70–80% for 2 weeks. The remaining plants were transferred to control conditions (C) into a greenhouse chamber at 20 °C day and 16 °C night temperature with a day length of 16 h at a light intensity of 200 μmol m^−2^ s^−1^. Control plants remained under these conditions until seed harvest. After cold priming, cold-treated plants were moved to control conditions for 7 days (lag phase), where half of these plants remained until seed harvest. The other half was triggered for 2 weeks by a second cold treatment (PT) under the same conditions as before. Then plants were transferred back to control conditions, where they remained until seed harvest (Suppl. Fig. S1). As in field experiments, inflorescences of all plants per pot were bagged for seed production and later assessment of seed yield by weighing.

### Climate data

Climate data for field experiments were obtained from a long-term meteorological station of the Potsdam Institute for Climate Impact Research (PIK) (https://www.pik-potsdam.de/de/produkte/klima-wetter-potsdam/klimazeitreihen). The following data were used: minimum temperature 5 cm above the ground (°C), daily sunshine duration (min), snow cover and precipitation (mm).

### Visual phenotyping

In March/April of the respective year field-grown plants were visually scored for absolute number (winter survival) and phenological developmental stage. The developmental stage was determined according to the Biologische Bundesanstalt, Bundessortenamt und Chemische Industrie (BBCH) scale (Meier 1997) adopted for *A. thaliana* (Schwachtje et al. 2011). The non-numeric BBCH scores were ranked in Excel with the function Rank.avg and further statistical analysis done in RStudio.

Plants from field experiments 2017/18 and 2018/19 and from all greenhouse experiments were continuously visually monitored to determine the exact date of bolting. These data were used for calculation of the phenological trait bolting time (BT), the number of days between sowing and differentiation of the inflorescence from the leaves at a size < 5 mm as previously described (Brachi et al. 2012). Five replicates were scored for each accession and condition in all experiments.

### Seed harvest and weighing

After harvesting, seeds were weighed to obtain the total seed yield per box (field) or pot (greenhouse) for each accession and condition. Seed mass per plant was obtained by dividing the total seed mass by the number of survivors per box or by the number of plants grown in one pot. 1000 seeds of each accession and replicate were counted at the Institute of Applied Genetics at the Free University of Berlin with an Elmor C3 seed-counting machine (Elmor AH). 1000 seed mass was weighed with a Sartorius LE244S precision balance (240 g × 0.0001 g) (Sartorius, Göttingen, Germany).

### Statistical analysis

R (R Development Core Team 2018) (version 4.0.3) and RStudio (R Development Core Team 2018) (version 1.3.1093) were used for statistical analysis. For correlation analyses, Pearson or Spearman correlation was used, depending on the scale level of the analysed variables (Schäfer and Schöttker-Königer 2015).

## Results

### Overview of field experiments and experiments under controlled conditions

**Fig. 1:**
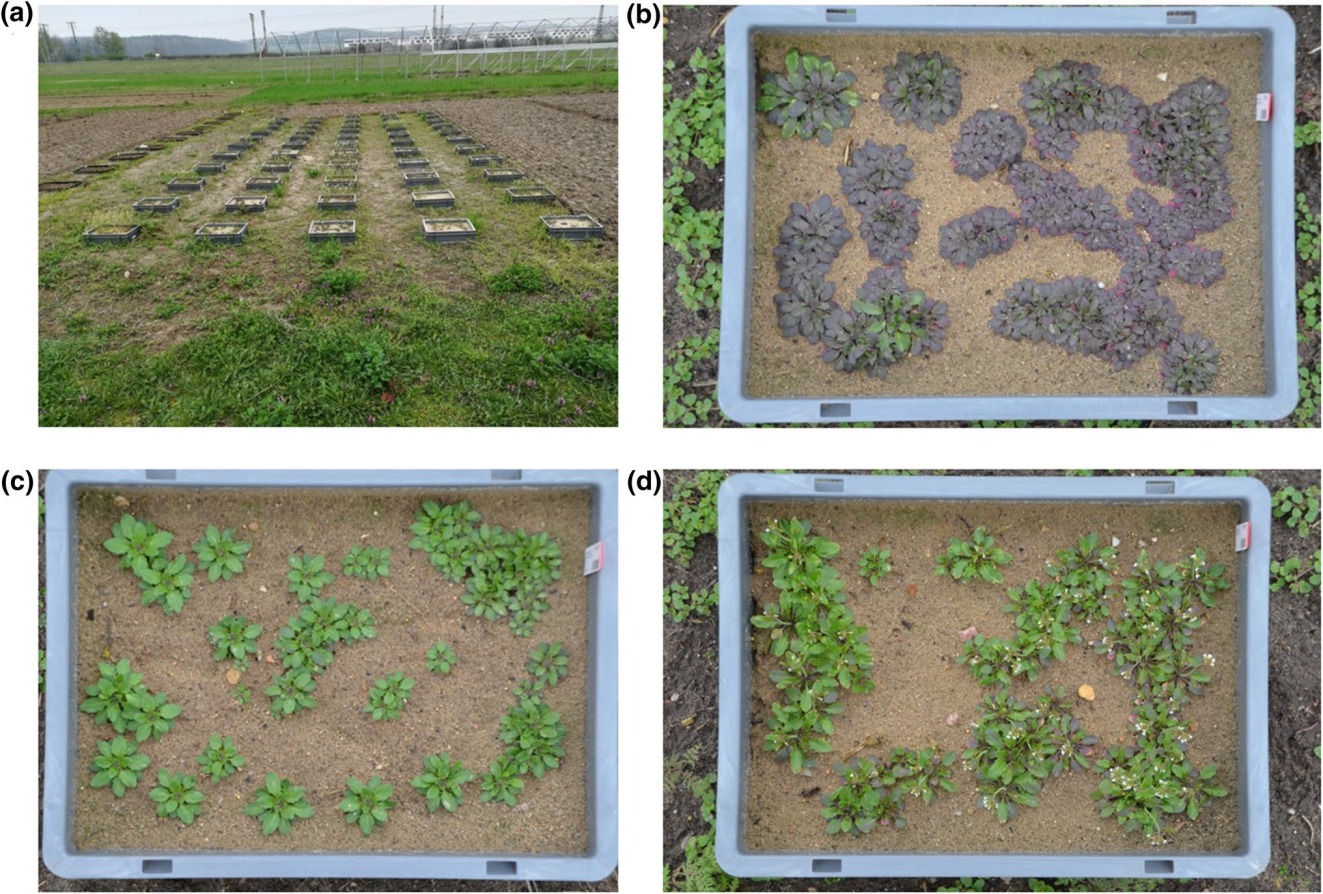
Photographs of the field trial 2018/19. **a** Overview of the experimental setup. Five rows with 11 boxes in a randomized design. **b** *A. thaliana* accession Col-0. **c** *A. thaliana* accession N13. **d** *A. thaliana* accession C24, flowering. Photos were taken on the field in Potsdam-Golm on the 15 of March 2019. The sowing was on the 30th of October 2018

**Fig. 2:**
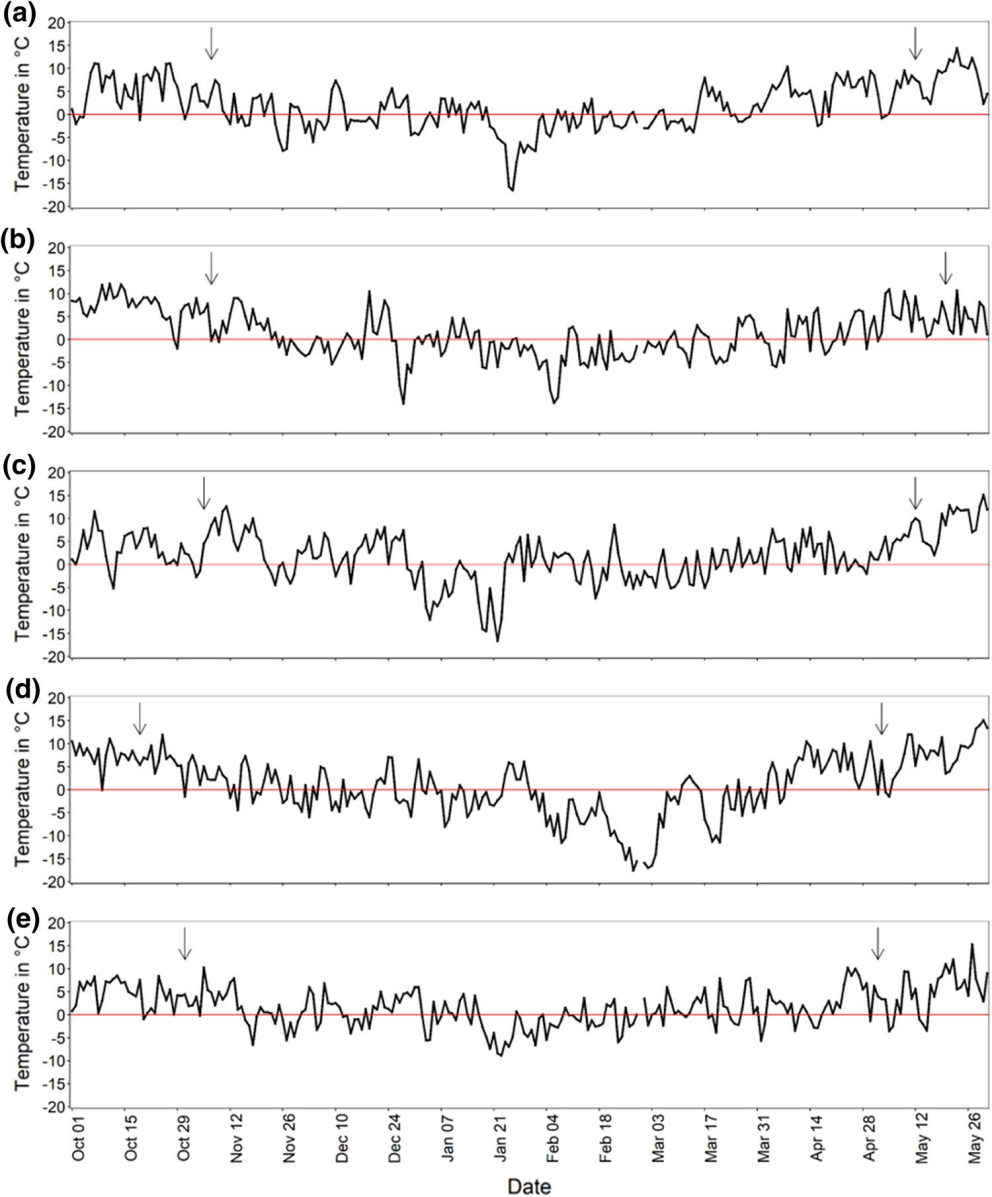
Daily minimum temperature near ground from October to the end of May for five different field experiments. Temperature was measured 5 cm above the ground in 2013/14 (**a**), 2014/15 (**b**), 2015/16 (**c**), 2017/18 (**d**) and 2018/19 (**e**). Climate data for field experiments were obtained from the long-term meteorological station of the Potsdam Institute for Climate Impact Research (PIK) (https://www.pik-potsdam.de/de/produkte/klima-wetter-potsdam/klimazeitreihen). The sowing date and the date of the first bagging of the inflorescences are marked with arrows

Five field trials were performed in 2013/14, 2014/15, 2015/16, 2017/18 and 2018/19 on a field in Potsdam–Golm (Germany). Eleven *A. thaliana* accessions (Suppl. Table S1) with different cold-acclimated freezing tolerance (expressed as LT_50_ACC) were grown from October to May the following year to investigate the effects of cold conditions on the fitness of different genotypes of the same plant species under natural conditions (Fig. 1). The average minimum temperature at 5 cm above the ground was relatively similar over the experimental period for all 5 years, with the highest in 2015/16 (0.8 °C) and the lowest in 2017/18 (− 0.5 °C) (Table 1, Suppl. Table S4-1). The average minimum temperature at 5 cm above the ground 2 months before bagging varied more with 3.8 °C in 2013/14 versus 0.7 °C in 2017/18, representing the highest and lowest values. On the contrary, the absolute minimum winter temperatures at 5 cm above the ground were very different, with the lowest temperature in 2017/18 (− 17.6 °C) and the highest in 2018/19 (− 8.9 °C). Generally, several longer strong frost events were recorded per year, except for 2018/19, with an extraordinarily mild winter (Fig. 2). The number of days with snow cover differed greatly in the different years, with 20 days in 2014/15 down to only 5 days in 2018/19. The average daily sunshine duration was the shortest in 2017/18 (208 min/day) and the longest in 2013/14 (230 min/day). Precipitation with 1.1–1.5 mm/day and relative humidity with a daily average of 78.1–81.6% were relatively similar for all years (Table 1).

**Table 1:** Weather parameters for all field trials

	07.11.2013–12.05.2014	07.11.2014–20.05.2015	05.11.2015–12.05.2016	19.10.2017–03.05.2018	31.10.2018–02.05.2019
Snow cover (days)	17	20	18	9	5
Sunshine duration (average min/day)	230 ± 16.7	224.3 ± 17.2	216 ± 17.6	208 ± 16.7	216.4 ± 18.2
Precipitation (average mm/day)	1.1 ± 0.2	1.1 ± 0.1	1.4 ± 0.2	1.5 ± 0.2	1.2 ± 0.2
Relative humidity (daily average %)	81.6 ± 0.8	79 ± 1.0	79.3 ± 0.9	81.4 ± 0.9	78.1 ± 1.1
Average minimum temperature 5 cm above ground (°C)	0.6 ± 0.3	0.0 ± 0.3	0.8 ± 0.4	− 0.5 ± 0.4	0.6 ± 0.3
Average minimum temperature 5 cm above ground two months before bagging (°C)	3.8 ± 0.5	2.2 ± 0.5	2.1 ± 0.4	0.7 ± 0.8	1.9 ± 0.5
Absolute minimum winter temperature 5 cm above ground (°C)	− 16.5	− 14.0	− 16.6	− 17.6	− 8.9

Averages are shown ± SE, if appropriate. Climate data for field experiments were obtained from a long-term meteorological station of the Potsdam Institute for Climate Impact Research (PIK) (https://www.pik-potsdam.de/de/produkte/klima-wetter-potsdam/klimazeitreihen)

Experiments under controlled climate conditions were performed in the years 2015, 2018 and 2019. In each experiment, five (2015) or six (2018 and 2019) natural accessions of *A. thaliana*, two with high, two with intermediate and one or two accessions with low cold-acclimated freezing tolerance (expressed as LT_50_ACC), overlapping with accessions investigated in the field trials, were grown. One-third of the plants were kept under control conditions (C), another third was cold treated (primed—P) for 14 days at 4 °C and the last third was after the first cold treatment exposed to 1 week of control conditions (lag phase) before getting cold treated (triggered—PT) again for 2 weeks at 4 °C (Suppl. Fig. 1). After triggering, all plants were kept under the same control conditions in a long-day greenhouse chamber at 20 °C till seed development. These conditions were chosen to resemble temperature fluctuations under field conditions.

### Bolting time differs between experiments under field and controlled conditions

**Fig. 3:**
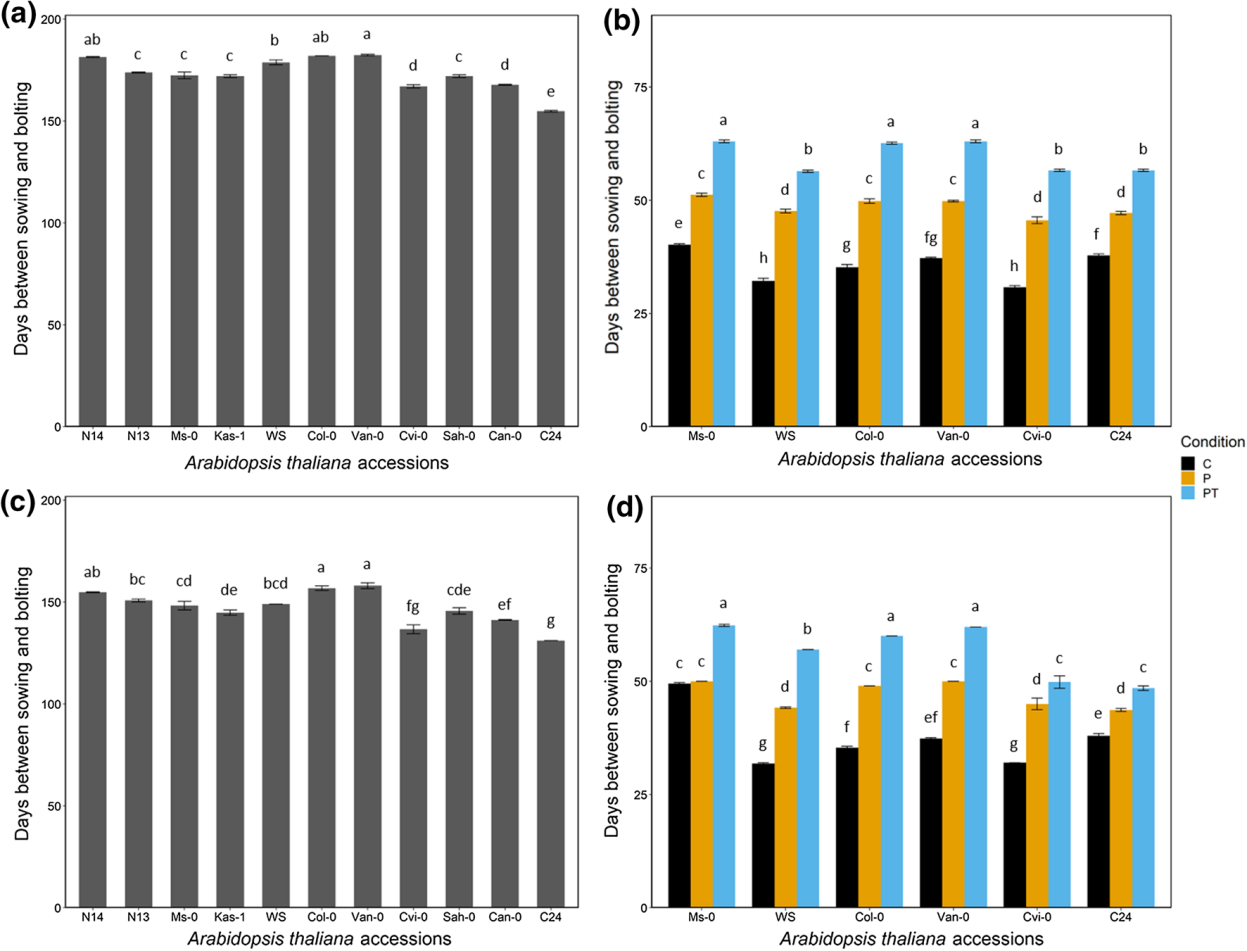
Comparison of bolting times in days between plants grown in the field trial 2017/18 (**a**) and 2018/19 (**c**) and those grown in the greenhouse experiment 2018 (**b**) and 2019 (**d**). Bars represent the average of five biological replicates ± SE. C, control; P, primed plants exposed to cold stress (4 °C) for two weeks; PT, primed and triggered plants exposed to cold stress (4 °C) for two weeks twice, with a lag phase of one week under control conditions in between both treatments. Accessions are ordered from the most tolerant (N14/Ms-0) to the most sensitive (C24) to freezing

Arabidopsis accessions are divided in winter and summer annuals, but transitions between these types are known depending on the temperature conditions (Wilczek et al. 2009) and under the here described conditions all accessions performed as winter annuals. Winter annual plants bolt and flower in spring after germination in autumn and overwintering. Under field conditions, the bolting time was in general shorter in 2018/19 compared to 2017/18 (Fig. 3, Suppl. Table S4-2). Bolting times differed greatly between cultivations when compared between two field and all greenhouse experiments. A direct comparison of bolting times between field and greenhouse experiments is shown exemplarily in Fig. 3. In the field trial 2017/18 and 2018/19 (Fig. 3a, c), plants needed between 131 and 184 days for the development of an inflorescence, with the shortest bolting time for the most freezing-sensitive accessions C24 or Can-0 and the longest bolting times for three accessions with either intermediate (Van-0, Col-0) or highest freezing tolerance (N14). In the greenhouse experiments 2018 and 2019 (Fig. 3b, d), the bolting time was clearly influenced by the extent of cold treatment with the shortest bolting times for the control (30–50 days) and the longest for PT plants (46–64 days) (Suppl. Table S5-1). The three accessions WS, Cvi-0 and C24 (except under control) had under all conditions shorter bolting times than the accessions Ms-0, Col-0 and Van-0.

### Field experiments

#### Winter survival and developmental stage correlate with temperature under field conditions

Winter survival, analysed in March after the cold period in winter, varied strongly between accessions and years (Suppl. Table S4-3) ranging from 3% up to 93% in single replicates. In 2013/14, survival for single replicates ranged from 20% (Sah-0) to 83% (Ms-0), in 2014/15 from 25% (Van-0) to 93% (N13), in 2015/16 from 3% (Can-0) to 78% (Ms-0), in 2017/18 from 22% (Van-0) to 82% (Col-0) and in 2018/19 from 8% (C24) to 90% (N13).

**Fig. 4:**
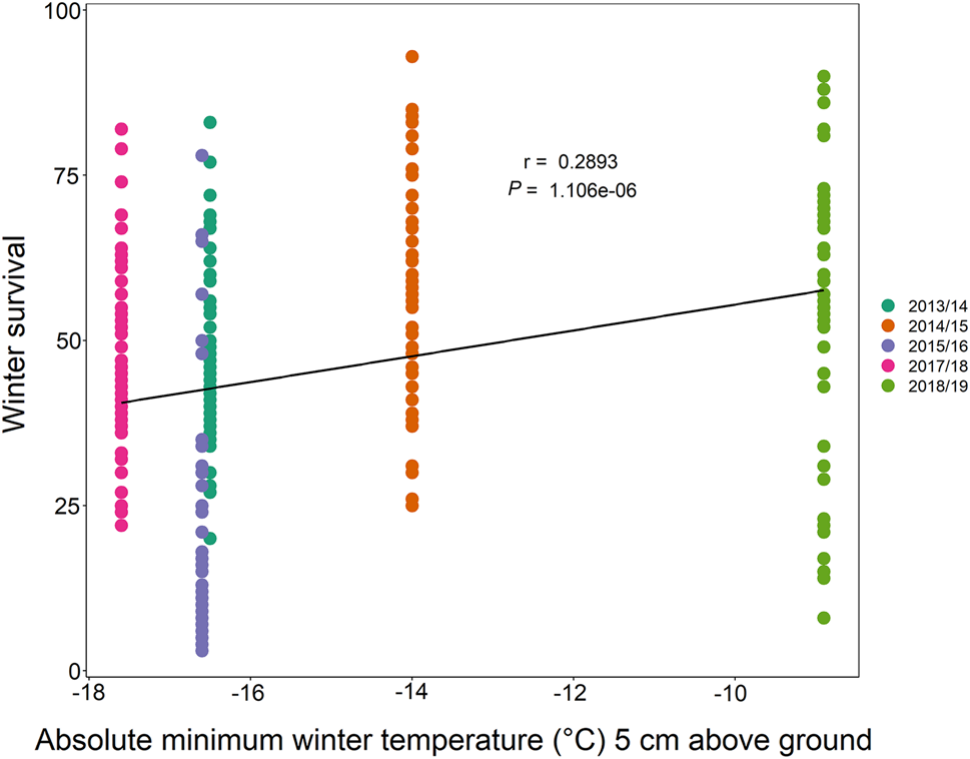
Correlation analysis of the effect of the absolute minimum winter temperature (°C) on the winter survival in 11 *Arabidopsis thaliana* accessions under field conditions. Winter survival was scored in 2013/14 (turquoise), 2014/15 (orange), 2015/16 (violet), 2017/18 (pink) and 2018/19 (green) for all accessions and plotted against the average of the absolute minimum daily temperature at 5 cm above ground (°C) during the respective experiment. Data points represent five replicates for each accession and year (*n* = 274). Pearson correlation coefficient (*r*) and *p* value are indicated. Accessions with description are listed in Table S1

**Fig. 5:**
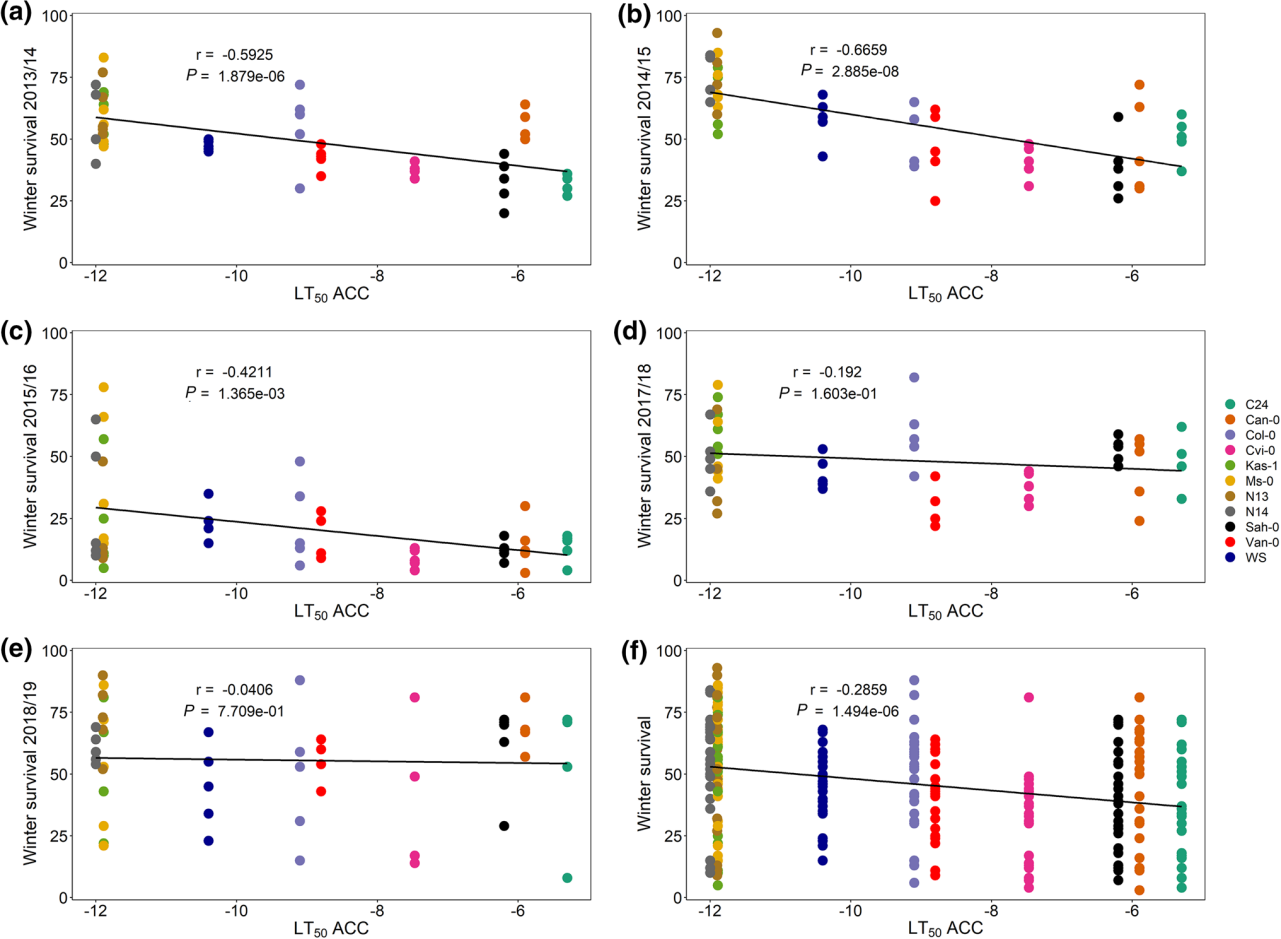
Correlation analysis of the effect of cold-acclimated freezing tolerance (LT_50_ ACC) on winter survival of 11 accessions of *A. thaliana* under field conditions. Winter survival was scored in the five field trials [2013/14 (**a**), 2014/15 (**b**), 2015/16 (**c**), 2017/18 (**d**), 2018/19 (**e**), all 5 years combined (**f**)]. Data points represent five biological replicates for each accession (differently coloured) (*n* = 55 for **a**–**d**, *n* = 54 for **e**; *n* = 274 for **f**). Pearson correlation coefficients (*r*) and *p* values are indicated. Accessions with description as well as their respective freezing tolerance (LT_50_ ACC) are listed in Table S1

The winter survival of all replicates of the 11 *A. thaliana* accessions correlated positively with the absolute minimum winter temperature at 5 cm above the ground in the five respective years (Fig. 4) and in addition with the number of days below 0 °C (Suppl. Fig. S2). A lower number of plants survived with decreasing absolute minimum winter temperature. When winter survival for all accessions was correlated separately with the absolute minimum winter temperature, a significant positive correlation was observed for four of them, three accessions with low freezing tolerance C24, Can-0, and Sah-0, and one with high freezing tolerance N13 (data not shown). Following this finding, the winter survival in each field trial was correlated with the cold-acclimated freezing tolerance (LT_50_ACC) previously determined for plants grown under controlled conditions (Zuther et al. 2012, 2015) (Fig. 5). The strongest negative correlation between these two parameters was obtained for the field trial 2014/15 (Fig. 5b), followed by 2013/14 (Fig. 5a) and 2015/16 (Fig. 5c). In the trials of 2017/18 and 2018/19, no significant correlation was found between winter survival and freezing tolerance. When combining all field trials, a significant negative correlation was still present (Fig. 5f), representing an improved winter survival for plants with higher freezing tolerance (lower LT_50_ACC).

As expected, the rank of the developmental stage of plants in the field trials (Suppl. Table S4-4), assessed as BBCH index (Suppl. Table S3), was strongly positively correlated with the average minimum temperatures (°C) over the 2 months preceding BBCH scoring (Suppl. Fig. S3). Furthermore, every accession used in the field trials showed a significant positive correlation (*P* < 0.05) between the average minimum temperature at 5 cm above ground in the 2 months preceding BBCH scoring (°C) and the ranked BBCH index over the five field trials in separate correlation analyses (data not shown).

### 1000 seed mass correlates with average minimum temperature and freezing tolerance

**Fig. 6:**
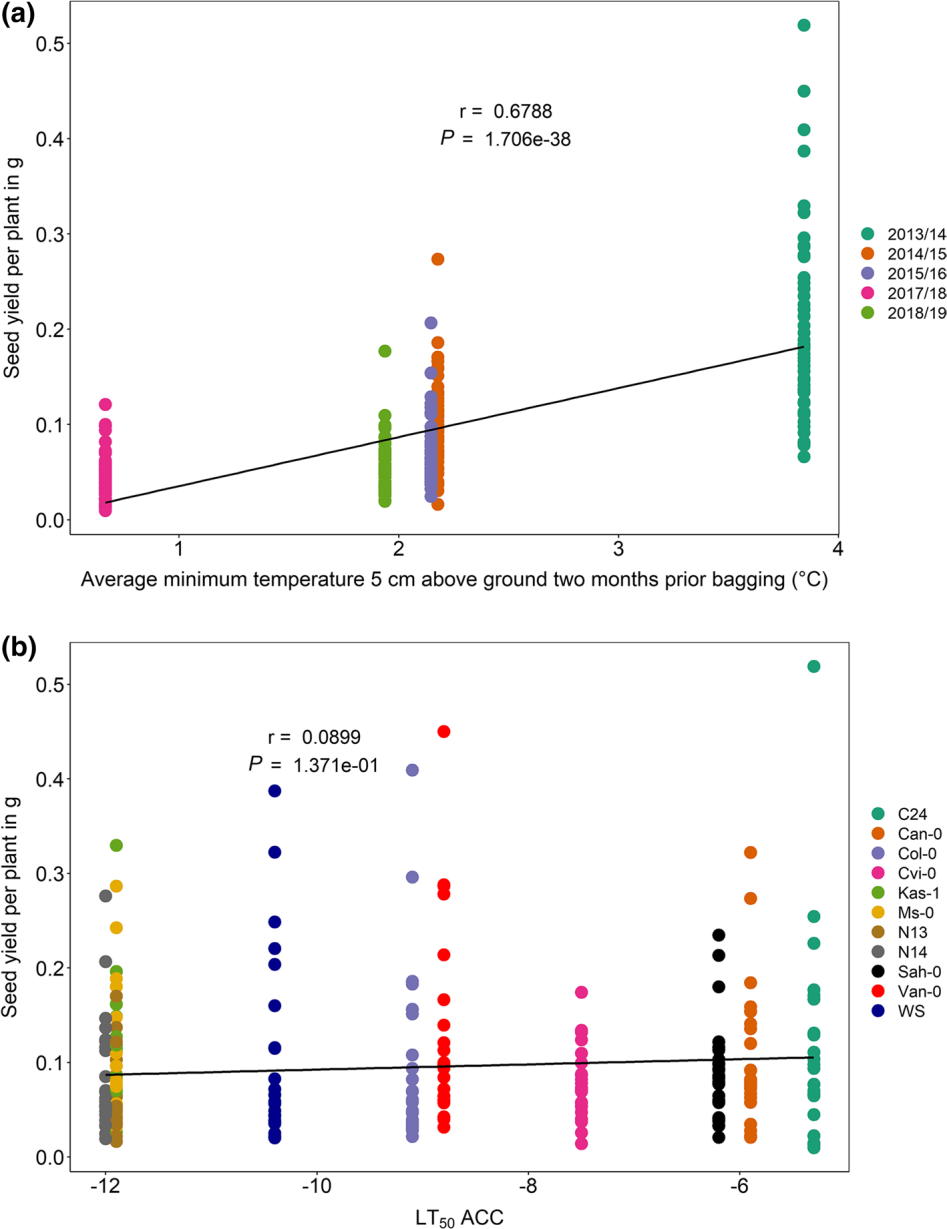
Correlation analyses of the effect of temperature before bagging and freezing tolerance as LT_50_ ACC on seed yield per plant in 11 *Arabidopsis thaliana* accessions under field conditions. Seed yield per plant (g) was calculated in 2013/14, 2014/15, 2015/16, 2017/18 and 2018/19 for all accessions and plotted against the average minimum temperature at 5 cm above ground (°C) in the 2 months prior to bagging of the plants (**a**) and the cold-acclimated freezing tolerance of each accession (**b**). Data points represent five replicates for each accession and year (*n* = 274). Pearson correlation coefficients (*r*) and *P* values are indicated. Accessions with description as well as their respective freezing tolerance (LT_50_ ACC) are listed in Table S1

Average seed yield per plant under field conditions ranged from 0.0163 g per plant for C24 in 2017/18 to 0.3033 g per plant for Van-0 in 2013/14 (Suppl. Table S4-5). The average seed yield per plant over all accessions was with 0.0442 g, the lowest in the 2017/18 field experiment with the lowest sunshine duration and the lowest average minimum temperature 5 cm above ground. The highest average seed yield per plant over all accessions was with 0.2014 g reached in 2013/14, the experiment with the longest sunshine duration and highest average minimum temperature 5 cm above ground 2 months before bagging (Suppl. Table S4-5 and Table 1). To investigate if the fitness of plants expressed as seed yield was affected by temperature, correlation analyses of the seed yield per plant with the average minimum temperature near the ground 2 months before bagging and with the cold-acclimated freezing tolerance of the respective accessions were done. Seed yield per plant was significantly positively correlated with the average minimum temperature at 5 cm above ground in the 2 months preceding bagging over all five field experiments (Fig. 6a). The higher the average minimum temperature in the 2 months preceding bagging, the wider was the dispersion of the data points. No correlation was found between the seed yield per plant (g) and the acclimated freezing tolerance (LT_50_ACC) of the respective accession (Fig. 6b). In a separate analysis of each of the 5 years, LT_50_ACC and seed yield per plant were only weakly correlated in the experiment of 2014/15 (data not shown).

**Fig. 7:**
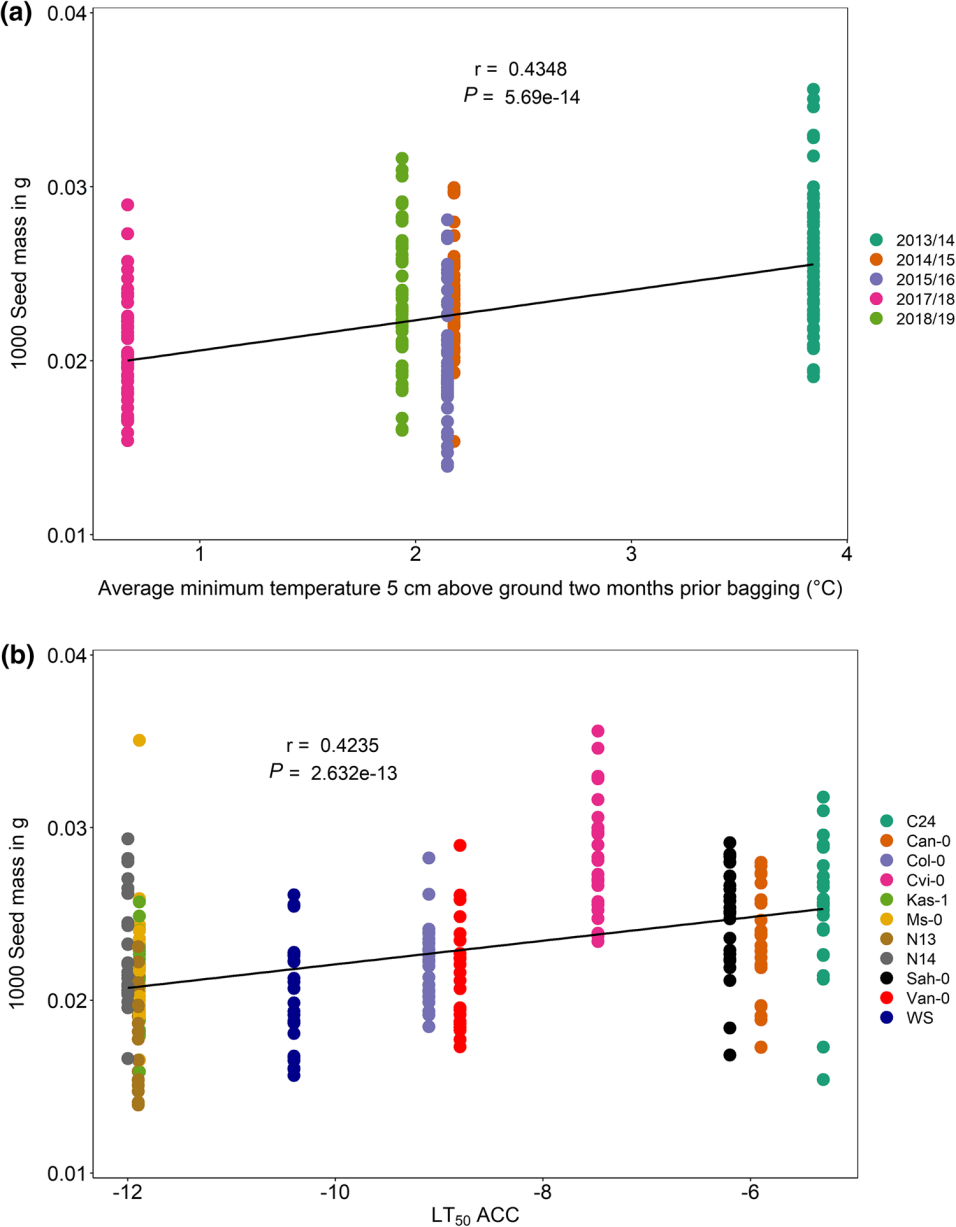
Correlation analyses of the effect of temperature before harvest and freezing tolerance as LT_50_ ACC on the mass of 1000 seeds in 11 *Arabidopsis thaliana* accessions under field conditions. 1000 seed mass (g) was determined for seeds harvested in 2013/14, 2014/15, 2015/16, 2017/18 and 2018/19 for all accessions and plotted against the average temperature at 5 cm above ground (°C) in the 2 months prior bagging of the plants (**a**) and the cold-acclimated freezing tolerance of each accession (**b**). Data points are from five replicates for each accession and year (*n* = 273). Pearson correlation coefficients (*r*) and *P* values are indicated. Accessions with description as well as their respective freezing tolerance (LT_50_ ACC) are listed in Table S1

The seed mass is another indicator for plant fitness. 1000 seeds were counted with an Elmor C3 seed-counting machine and the 1000 seed mass calculated for all experiments (Suppl. Table S4-6). While 1000 seed mass differed between the years, N13 was the accession with the lowest and Cvi-0 the one with the highest 1000 seed mass in all field trials (Suppl. Fig. S4). Subsequently, correlation analyses were performed to test for significant correlations (*P* < 0.05) between the 1000 seed mass and the average minimum temperature at 5 cm above the ground or the acclimated freezing tolerance (LT_50_ACC) of the accessions. Both parameters were positively correlated with the 1000 seed mass (Fig. 7), revealing higher seed mass with higher temperature above the ground and for accessions with lower freezing tolerance (higher LT_50_ACC). When correlations were performed for each of the 4 years separately, the analysis resulted in a significant positive correlation between LT_50_ ACC and the 1000 seed mass in all cases (Suppl. Fig. S5).

### Correlations of 1000 seed mass with freezing tolerance were confirmed in controlled chamber experiments under three different conditions

In three greenhouse experiments in 2015, 2018 and 2019, seed yield was measured for five to six accessions, overlapping with accessions investigated under field conditions. Here, the accessions were grown under three different conditions: control conditions (C), cold primed (P) and cold primed and triggered (PT) (Suppl. Fig. S1). The lowest averaged seed yield per plant was determined in the accession Ms-0 under control conditions in all three experiments and the highest in C24 after PT (2015) (Suppl. Fig. S6; Suppl. Table S5-2). A consistent significant increase in seed yield after cold priming (P) was found in at least two experiments for Ms-0 and Van-0. After cold priming and triggering (PT), the seed yield of the accessions Ms-0, Van-0 and C24 was higher in at least two experiments compared to the control.

**Fig. 8:**
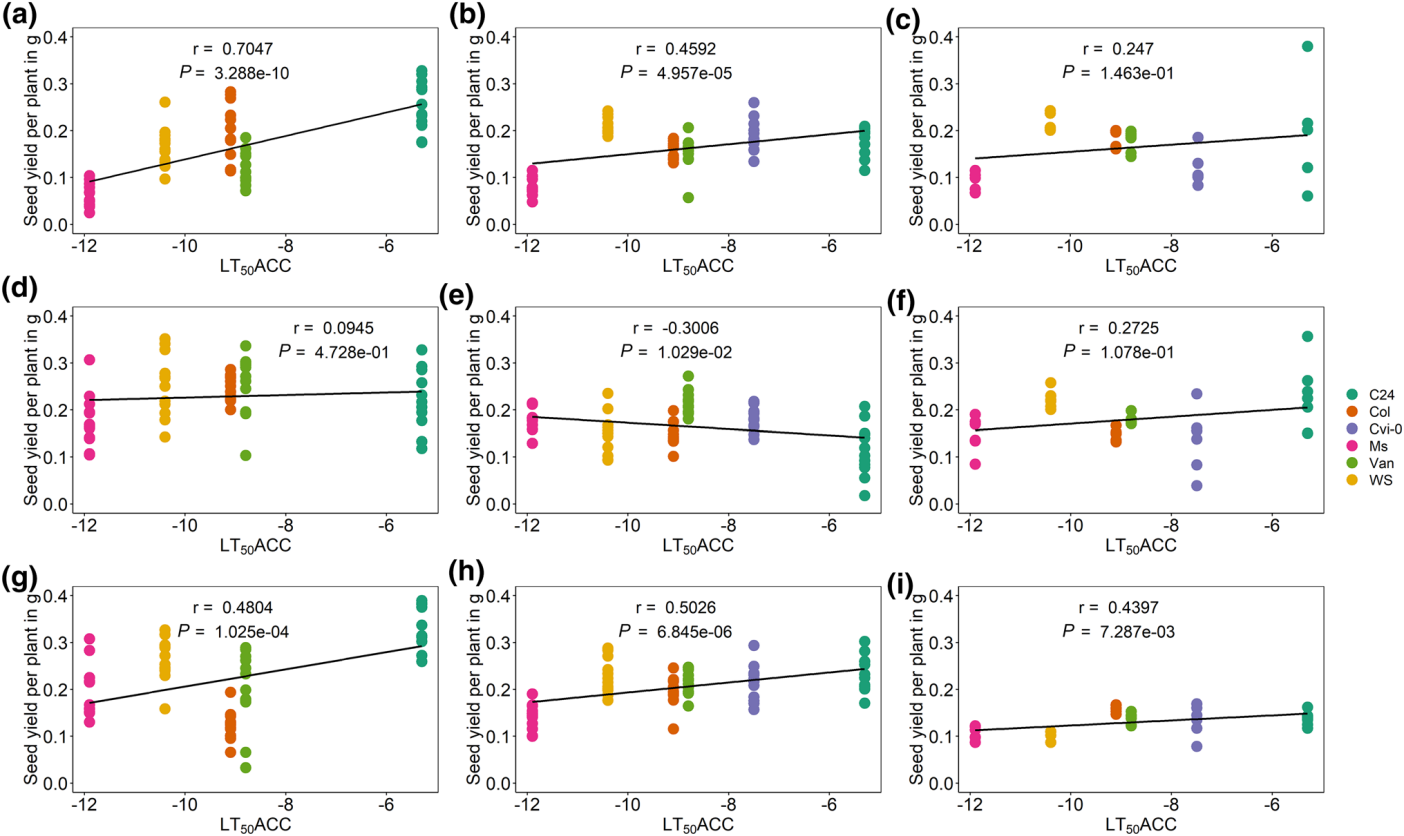
Correlation analysis of the effect of freezing tolerance (LT_50_ ACC) on the seed yield per plant of different *A. thaliana* accessions grown in the greenhouse under three different conditions. **a, d, g** Experiment 2015 with five accessions. **b, e, h** Experiment 2018. **c, f, i** Experiment 2019 with six accessions. **a, b, c** Control group, grown under long-day conditions; C, control. **d, e, f** Primed group exposed to cold stress (4 °C) for 2 weeks; P, priming. **g, h, i** Primed and triggered group exposed to cold stress (4 °C) for 2 weeks twice, with an interval of 1 week under control conditions in between both treatments; PT, priming and triggering. Data points are from 12 replicates for each accession and condition for experiments 2015 and 2018 and from six replicates for experiment 2019. Pearson correlation coefficients (*r*) and *P* values for the linear regression are indicated. Accessions with description as well as their respective freezing tolerance (LT_50_ ACC) are listed in Table S1

Seed yield per plant (g) was positively significantly correlated with the freezing tolerance of acclimated plants (LT_50_ACC) in plants grown under control conditions in 2015 and 2018 (Fig. 8a, b) and for primed and triggered plants (PT) in all 3 years (Fig. 8g–i). The seed yield of cold-primed plants instead did not significantly correlate with LT_50_ACC in 2015 and 2019 (Fig. 8d, f) and showed an even negative correlation in 2018 (Fig. 8e).

**Fig. 9:**
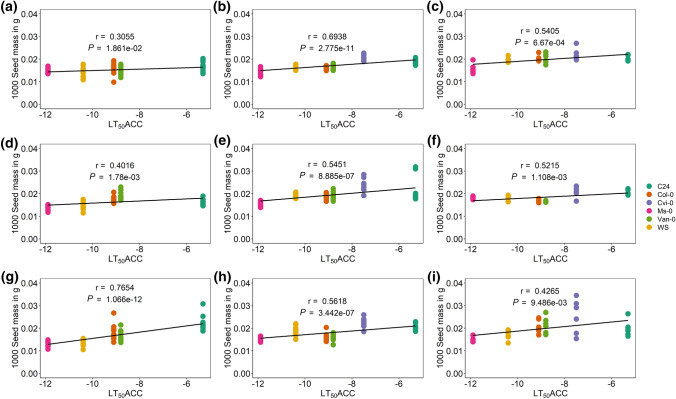
Correlation analysis of the effect of freezing tolerance (LT_50_ ACC) on the 1000 seed mass per plant of different *A. thaliana* accessions grown in the greenhouse under three different conditions. **a, d, g** Experiment 2015 with five accessions. **b, e, h** Experiment 2018. **c, f, i** Experiment 2019 with six accessions. **a, b, c** Control group, grown under long-day conditions; C, control. **d, e, f** Primed group exposed to cold stress (4 °C) for 2 weeks; P, priming. **g, h, i** Primed and triggered group exposed to cold stress (4 °C) for 2 weeks twice, with an interval of 1 week under control conditions in between both treatments; PT, priming and triggering. Data points are from 12 replicates for each accession and condition for experiments 2015 and 2018 and from six replicates for experiment 2019. Pearson correlation coefficients (*r*) and *P* values for the linear regression are indicated. Accessions with description as well as their respective freezing tolerance (LT_50_ ACC) are listed in Suppl. Table S1

For all controlled chamber experiments, 1000 seed masses were weighed and calculated for each accession and condition (Suppl. Fig. S7) The lowest 1000 seed mass was recorded for Van-0 under control conditions (2015), the most freezing-tolerant accession Ms-0 under P and PT in 2015 and for WS at PT in 2015 (Suppl. Table S5-3). The highest 1000 seed mass was detected in the freezing-sensitive accessions Cvi-0 at PT conditions (2019). No consistent significant changes in at least two experiments of the 1000 seed mass were recorded after cold priming (P) or cold priming and triggering (PT) compared to control. The 1000 seed mass was positively significantly correlated with the freezing tolerance of acclimated plants (LT_50_ACC) in all three experiments and for all three conditions (C, P, PT) (Fig. 9). The weakest correlation was observed for the 1000 seed mass of control plants in 2015 (Fig. 9a) and the strongest for the 1000 seed mass of cold-primed and triggered plants in 2019 (Fig. 9g).

## Discussion

Cold acclimation-related traits and freezing tolerance are important for the physiological adaptation of plants to cold environments (Zhen and Ungerer 2008; Thomashow 2010; Preston and Sandve 2013; Oakley et al. 2014). Different Arabidopsis populations across Europe and Asia cope with highly varying climate conditions (Lasky et al. 2012; Frachon et al. 2018; Price et al. 2020) resulting in local adaptation, shown for e.g. for populations from Sweden and Italy (Fournier-Level et al. 2011; Ågren and Schemske 2012). Cold acclimation resulting in the development of higher freezing tolerance is beneficial in autumn, winter and spring when temperatures are low and freezing events are likely; however, it might be costly, especially in spring when the balance between preservation of cold acclimation and subsequent deacclimation secures survival and timely reproduction, but also results in competition for metabolic resources. It is still unclear whether cold priming and preservation of a higher freezing tolerance entail a fitness cost or not.

To resolve this question, we performed five overwintering experiments in a field site outside of the MPI-MP in Potsdam during the seasons 2013/14, 2014/15, 2015/16, 2017/18 and 2018/19 with 11 accessions covering the whole freezing tolerance range of *Arabidopsis* (Zuther et al. 2012). With these long-term multiyear experiments, we covered responses to extreme weather events and variation between years and uncovered general rather than short-term response patterns.

### Phenological parameters were highly influenced by temperature

The developmental state as ranked BBCH index was positively correlated with the average minimum temperature near ground in the 2 months prior to scoring and in all field trials in separate correlation analyses. Consequently, the development of plants was strongly influenced by air temperature, as indicated before (Hatfield and Prueger 2015). That also means that climate change with warmer temperatures will advance reproductive plant development.

Bolting as initiation of flowering and transition from vegetative to reproductive growth is an important fitness-related morphological parameter for the reproductive success of a plant, as it influences seed production and therefore the survival of the species (Chen et al. 2019).

Bolting times were three to five times longer in field than in greenhouse trials. Bolting and later flowering are affected by long photoperiods and low non-freezing temperature (Chen et al. 2019). Longer bolting times can be explained by the fact that plants in the field were exposed to a long cold and short-day period from October to March when metabolic processes were slowed down and growth and development were mostly arrested. Only in spring, growth was resumed and the generative phase initiated, as indicated by inflorescence axis elongation. The decelerating influence of cold temperature is corroborated by differences in bolting times between the three temperature conditions for all accessions in greenhouse experiments. Control plants needed the least time to bolt, followed by P plants and PT plants. Bolting times differed also between different field experiments and were lower in 2018/19 with a higher average minimum temperature above ground and a higher absolute minimum winter temperature.

### Winter survival depended strongly on minimum temperatures and acclimated freezing tolerance in field experiments

Winter survival in five field trials over all accessions was linearly positively correlated with the absolute minimum winter temperatures 5 cm above the ground for the respective years. When the accessions were considered separately, only three freezing-sensitive and one freezing-tolerant accession showed positive correlations. Another study found that the establishment rates (i.e. survival rates) of *A. thaliana* in field experiments varied greatly among different genotypes and in different years (Köhl and Laitinen 2015). In replicated common garden experiments at different field sites in Europe with a geographically diverse set of inbred lines from natural populations, alleles assigned to survival were described to be mainly influenced by temperature (Fournier-Level et al. 2011).

Winter survival was lowest in 2017/18 with the lowest average minimum temperature and the absolute minimum winter temperature 5 cm above ground and additionally two cold spells after partial deacclimation in March with unusual freezing temperatures. It was highest in 2018/19, the period with the smallest temperature fluctuations and the most warm winter temperature 5 cm above the ground.

Alternating warm and freeze temperatures have negative effects on plants winter survival, but the vulnerability of plants to erratic temperatures depends on their frost hardiness and deacclimation kinetics (Pagter and Arora 2013). Following that, we could show that the winter survival of *A. thaliana* accessions was strongly correlated with their respective freezing tolerance after cold priming, determined as LT_50_ in electrolyte leakage assays under controlled conditions (Zuther et al. 2012), over all field trials. A slower deacclimation rate in more freezing-tolerant accessions compared to sensitive ones might have had an additional positive influence on survival (Zuther et al. 2015). A high importance of cold acclimation capacity was also found for forage grasses with a high correlation of freezing tolerance and winter survival in the field (Rognli 2013). For Northern American Arabidopsis populations, lower survival of more distant originating lines when moving north was found suggesting that the climatic history of a population predicts the phenotype (Samis et al. 2019).

However, the winter survival of *A. thaliana* accessions was not correlated with acclimated freezing tolerance when considering the field trials in 2017/18 and 2018/19 separately. This might be due to extraordinary weather conditions during these two winters. In the first three trials with significant correlations, strong frost was recorded from December to February, favouring more freezing-tolerant accessions, whereas in the two last trials, only mild frost was recorded during winter with the first severe frost event occurring only at the end of February (2017/18) or not at all (2018/19).

In conclusion, winter survival depended both on the absolute minimum winter temperature and the acclimated freezing tolerance of plants, but there might be still other factors that could have had an influence on winter survival such as temperature oscillations, precipitation or snow cover.

### Cold survival is cost intense, as fitness was reduced with lower temperature

We showed that the general fitness expressed as total seed weight and the 1000 seed mass were positively correlated with the average minimum temperature above the ground in the 2 months preceding bagging over all five field trials. Also in an experiment with 279 Arabidopsis accessions on the Iberian Peninsula with Mediterranean climate, seed weight correlated significantly with the annual mean temperature (Manzano-Piedras et al. 2014). Local adaptation, defined as higher relative fitness of a local compared to a foreign genotype (Oakley et al. 2014), might have influenced the seed yield. Nevertheless, accessions from more distant origins (Cvi-0, Can-0) showed rather higher seed yield than Ms-0 from Moscow or Col-0 from Poland. When the fitness of Arabidopsis accessions was tested as adaptation to warmer climates in a four site common garden experiment across Europe, genotypes from warmer regions than the experimental site had higher relative fitness compared to local genotypes in all four sites (Wilczek et al. 2014), which is similar to our findings. Higher productivity of freezing-sensitive accessions originating from lower latitudes might have also been caused by longer silique length found in accessions from lower latitudes by the evaluation of 204 geoclimatic variables from 1131 local environments of Arabidopsis (Ferrero-Serrano and Assmann 2019).

The strong correlation between seed yield and the average minimum temperature above ground in the 2 months preceding bagging in field experiments implies that long-term adaptation to lower temperatures including cold survival generates some cost for the plants.

These costs were not to be seen when the cold treatment was done under controlled conditions including repeated short-term cold treatments. Several reasons might account for this difference, such as the lack of overwintering, an only short-term cold treatment, the missing exposure to temperatures below zero and the solely application of a single stress. When comparing the same stress resistance level, it is expected that fitness costs will be higher under resource-poor compared to optimal conditions as present under controlled conditions (Walters and Heil 2007). Consistent with our findings, no impact on seed number or yield was found comparing seven accessions under controlled conditions after a 2-week pre-cold treatment (Cvetkovic et al. 2017). Obviously, experiments under controlled conditions lack the complex weather and stress conditions present under natural conditions, e.g. the influence of UV light (Schulz et al. 2021). A cold pre-treatment of seven Arabidopsis accessions for 14 days at 4 °C under controlled conditions with a subsequent transfer to field conditions in March or May positively influenced the reproductive fitness in all accessions (Cvetkovic et al. 2017). When plants experienced additional cold in the field after the March transfer two freezing-sensitive accessions developed higher seed yield (Cvetkovic et al. 2017).

Recently, Nagano et al. (2019) investigated seasonal variation in the transcriptome dynamics of *Arabidopsis halleri* subsp. *gemmifera* in field experiments and compared the resulting fitness with plants grown in growth chambers simulating in-phase and anti-phase oscillations between temperature and day length. Higher fitness (flower production, numbers of opened flowers, leaf number) was recorded in the natural environment and temperature change was identified as a dominant factor influencing gene expression levels and thereby mostly explaining seasonal oscillations in gene expression (Nagano et al. 2019).

Higher phenotypic plasticity was shown in common garden experiments compared to experiments under controlled conditions, especially for a high-altitude population of *Arabidopsis thaliana* of the west Himalaya compared to low-altitude ones (Singh and Roy 2017). Other studies revealed a missing reproducibility of parameters raised in field and controlled chamber conditions, as e.g. for flowering time (Wilczek et al. 2009; Brachi et al. 2012; Mishra et al. 2012).

### An improved acclimated freezing tolerance is costly for the 1000 seed mass under natural as well as controlled conditions

Acclimated freezing tolerance (LT_50_ACC) and 1000 seed mass were positively correlated in the field trials as well as in the greenhouse fitness experiments across 3 years and all three conditions, pointing to a lower fitness in accessions with a higher freezing tolerance. Plants can increase their fitness by producing larger and/or heavier seeds, which support the embryo and the seedling with more resources. More freezing-tolerant accessions (northern accessions) displayed lower fitness (1000 seed mass) compared to more freezing-sensitive accessions (southern accessions) after overwintering or exposure to cold (Figs. 7, 9). Clinal variation was reported for the high genetic variability of Arabidopsis accessions for several traits, as e.g. winter survival (Montesinos et al. 2009) or biomass allocation and fecundity (Montesinos-Navarro et al. 2011). Clinal variation for freezing tolerance was shown for Arabidopsis (Zhen and Ungerer 2008; Zuther et al. 2012) in experiments under controlled conditions, for northern populations of *Arabidopsis lyrata* when collected along a latitudinal gradient (Wos and Willi 2015) or for trees grown in common garden trials (Holliday et al. 2010).

A common garden experiment with 21 Arabidopsis accessions in Maryland (USA) to test the influence of developmental parameters on plant fitness revealed climatic history as more influential for the performance compared to the actual life history within the experiment (Rutter and Fenster 2007). It was also previously recorded that accessions with an origin in environments characterized by extreme annual mean temperatures showed reduced fitness parameters (Manzano-Piedras et al. 2014). In addition, more frost-tolerant *A. lyrata* populations from higher latitudes depicted smaller plant size (Wos and Willi 2015). A correlation of seed yield (1000 seed mass was not measured) and cold acclimation potential was also found when Arabidopsis accessions were transferred after a cold pre-treatment to the field in May favouring accessions with intermediate and low cold acclimation potential (Cvetkovic et al. 2017).

Though cold acclimation is an inducible stress adaptation, it seems to be costly with even higher costs when the freezing tolerance is more highly expressed. A study that examined local adaptation during early life stages in *A. thaliana* in reciprocal transplant experiments found that genetically based traits affecting performance in young plants strongly influence adaptive differentiation and genetic trade-offs (Postma and Ågren 2016).

The fitness costs connected with increasing freezing tolerance are in accordance with the allocation-cost theories stating that growth and reproduction are limited by investing energy into defence strategies and acclimation (Herms and Mattson 1992; Walters and Heil 2007). Costs associated with higher tolerance to stress have been evidenced with plants constitutively expressing higher tolerance even in a non-stress environment. This is in accordance with our findings of a correlation between total seed yield and acclimated freezing tolerance in all three experiments under controlled conditions without any cold treatment. A constitutive *CBF* overexpression in Arabidopsis initiating a cold response under non-acclimated conditions resulted in growth retardation, flowering delay and seed yield reduction (Liu et al. 1998; Gilmour et al. 2000). Overexpression of CBF2 and CBF3, but not CBF1 reduced fitness (fruit number) expressed as growth and seed production under non-acclimated and acclimated conditions (Jackson et al. 2004). These fitness costs evoked by constitutive expression of the *CBF* genes could be eliminated when using inducible promoters (Kasuga et al. 1999; Lee et al. 2003).

A costly development of freezing tolerance and a fine-tuning between tolerance costs and benefits was suggested for a Swedish genotype when grown in Sweden (Oakley et al. 2014).

On the other hand, when comparing a recombinant inbred mapping population from locally adapted populations of *Arabidopsis thaliana* from Sweden and Italy in growth chamber experiments, all investigated photosynthesis-related traits possibly contributing to better growth were more exposed in the more freezing-tolerant Swedish line (Oakley et al. 2018). QTL associated with fitness under controlled conditions overlapped with fitness-based QTL previously identified in several experiments under natural conditions. A co-localization of QTL responsible for freezing tolerance differences with overall fitness QTL indicated the importance of freezing tolerance for local adaptation (Oakley et al. 2018). In addition, candidate SNPs with high absolute allele frequency differentiation between populations from Italy and Sweden were associated with fitness QT and linked freezing tolerance amongst other parameters to local adaptation (Price et al. 2020). Adaptation to varying environmental conditions can be reached by alterations in surprisingly few genomic regions and is often linked to fitness trade-offs (Ågren et al. 2013). When comparing seven previously identified freezing tolerance-related QTL with QTL identified for a Swedish accession, five QTLs for increased freezing tolerance were identified to co-localize. Three of these co-localized in addition to QTL identified for improved survival and fitness in Sweden favouring cold acclimation ability as the main reason for the trade-offs in nature (Oakley et al. 2014). In multiple natural environments with a large set of inbred lines from natural populations, it was shown that only 12 of 797 SNPs were related to fitness in more than one environment, which suggests that loci conveying large genetic effects are site independent (Fournier-Level et al. 2011). Local alleles related to high fitness were especially linked to climatic factors and underlined local adaptation in Arabidopsis (Fournier-Level et al. 2011).

These results suggest that there is a yield penalty expressed either as total seed yield reduction or smaller seed size associated with increasing freezing tolerance in plants grown under control conditions and in environments with recurring cold spells. The results of this study have to be validated through further long-term studies under natural and controlled conditions and in reciprocal transplant experiments with larger population size.

## Conclusions

Winter survival and developmental stage depended highly on temperature in long-term field experiments with 11 Arabidopsis accessions representing a wide range of cold acclimation capacity. Total seed yield per plant was affected by temperature with lower temperature yielding lower yield under field conditions, but not dependent on the cold acclimation capacity. Seed yield was not affected by temperature when low temperatures above 0 °C were applied. A higher acclimated freezing tolerance improved winter survival, but caused reductions in 1000 seed mass suggesting a fitness cost for the single seed, favouring seeds from more southern accessions at warmer temperatures. Consequently, proceeding climate change with rising temperatures might shift the distribution of more southern accessions to the north. Research in the model plant Arabidopsis should be complemented by investigations of fitness costs for crop plants for early detection of changing ecosystems and possible agricultural challenges.

### *Author contribution statement*

MB performed one field and one greenhouse experiment, seed counting and weighing and the data analyses for all experiments, EK performed two greenhouse experiments including phenotyping, seed weighing and counting, KK developed the general experimental design for Arabidopsis field experiments and adapted the BBCH score for Arabidopsis, MP performed several field experiments, EZ designed and performed all experiments and supervised the project. MB and EZ wrote the manuscript with contributions of all authors.

## Supplementary Information

Below is the link to the electronic supplementary material.

Supplementary file1 (DOCX 2140 KB)

Supplementary file2 (DOCX 29 KB)

Supplementary file3 (XLSX 49 KB)

Supplementary file4 (XLSX 42 KB)

## Data Availability

All data generated or analysed during this study are included in this published article and its supplementary information files.

## Abbreviations

ACC: Acclimated
BBCH: Biologische Bundesanstalt, Bundessortenamt und Chemische Industrie
C: Control
LT_50_: Temperature of 50% electrolyte leakage
P: Cold primed
PT: Cold primed and triggered
QTL: Quantitative trait locus
