# Supplementary material for: Cold stress and freezing tolerance negatively affect the fitness of Arabidopsis thaliana accessions under field and controlled conditions
Source: Planta. 2022 Jan 15;255(2):39. doi: 10.1007/s00425-021-03809-8 (PMC8761124; doi:10.1007/s00425-021-03809-8)
Supplement: Supplementary file 1 — Supplementary file1 (DOCX 2140 KB) [file 425_2021_3809_MOESM1_ESM.docx]

# **Cold stress and freezing tolerance negatively affect fitness of *Arabidopsis thaliana* accessions under field and controlled conditions**

# Maximilian Boinot^1^, Esra Karakas^1^, Karin Koehl^1^, Majken Pagter^1,2^, Ellen Zuther^1^

# ^1^Max Planck Institute of Molecular Plant Physiology, Am Muehlenberg 1, 14476 Potsdam, Germany

^2^Department of Chemistry and Bioscience, Aalborg University, 9220 Aalborg East, Denmark

*****Correspondence: [zuther@mpimp-golm.mpg.de](mailto:zuther@mpimp-golm.mpg.de)

Planta

# **Suppl. Figures:**


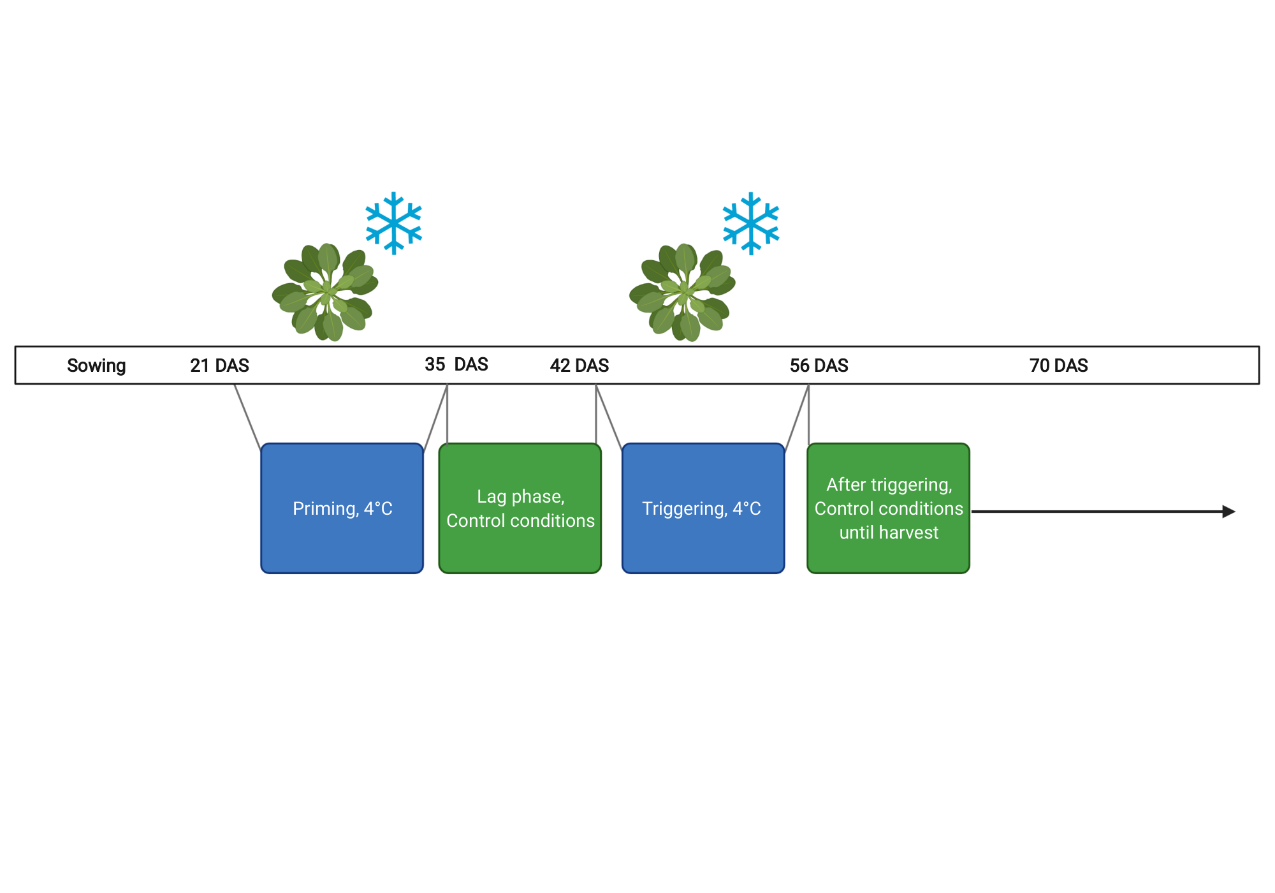


**Suppl. Fig. S1** Time course of the controlled-climate experiments. Control group, grown under long day conditions without any cold treatment; Primed group exposed to cold stress (4°C) for two weeks (priming) after three weeks of control conditions; Primed and triggered group exposed to cold stress (4°C) for two weeks twice, after three and six weeks respectively, with an interval of one week under control conditions (lag phase) in between both treatments (priming and triggering). Control conditions are coloured in green, cold treatments are marked in blue. DAS, days after sowing.

**
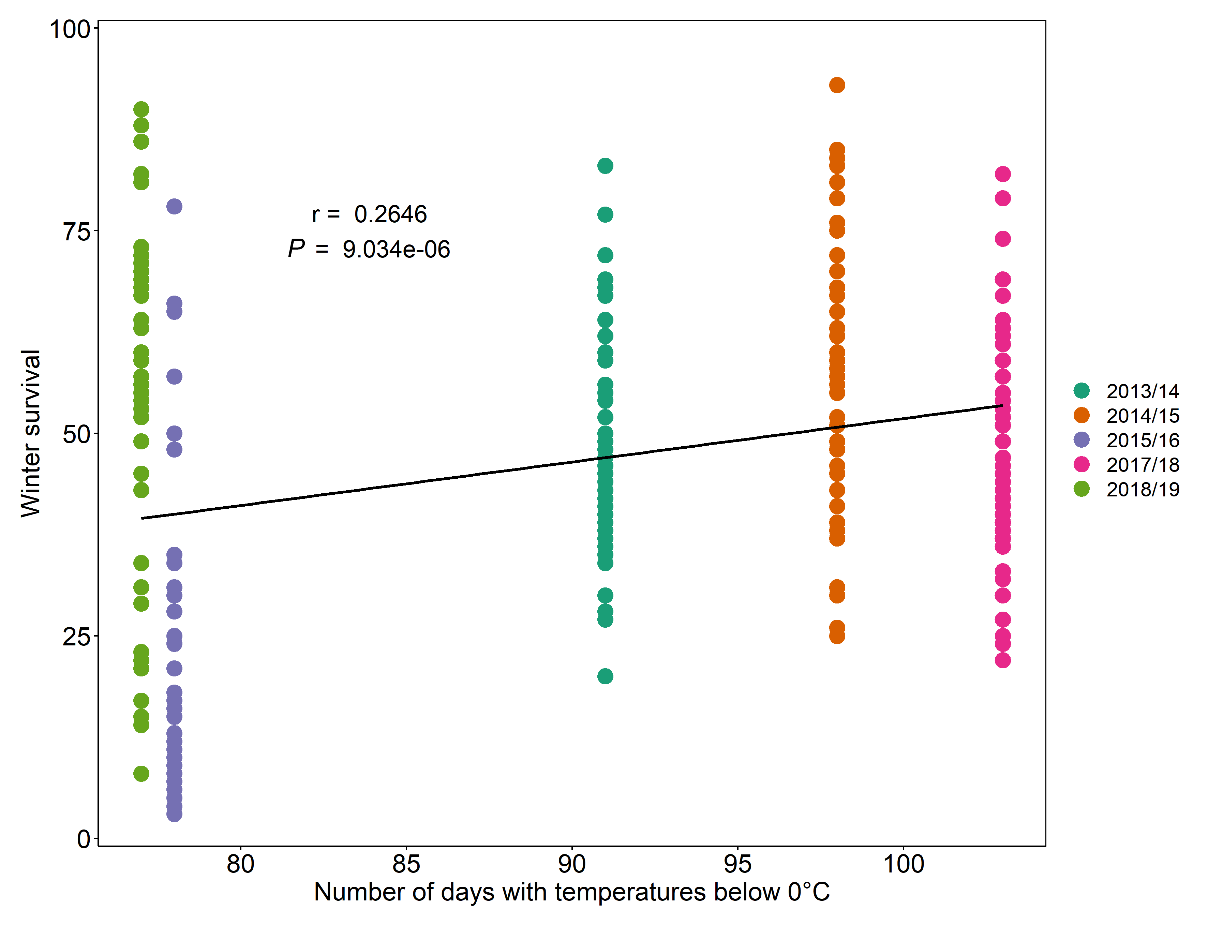
**

**Suppl. Fig. S2** Correlation analysis of the effect of the number of days below 0°C on the winter survival in 11 *Arabidopsis thaliana* accessions under field conditions. Winter survival was scored in 2013/14 (turquoise), 2014/15 (orange), 2015/16 (violet), 2017/18 (pink) and 2018/19 (green) for all accessions and plotted against the number of days below 0°C during the respective experiment. Data points represent five replicates for each accession and year. Pearson correlation coefficient (r) and *P*-value are indicated. Accessions with description are listed in Suppl. Table S1.


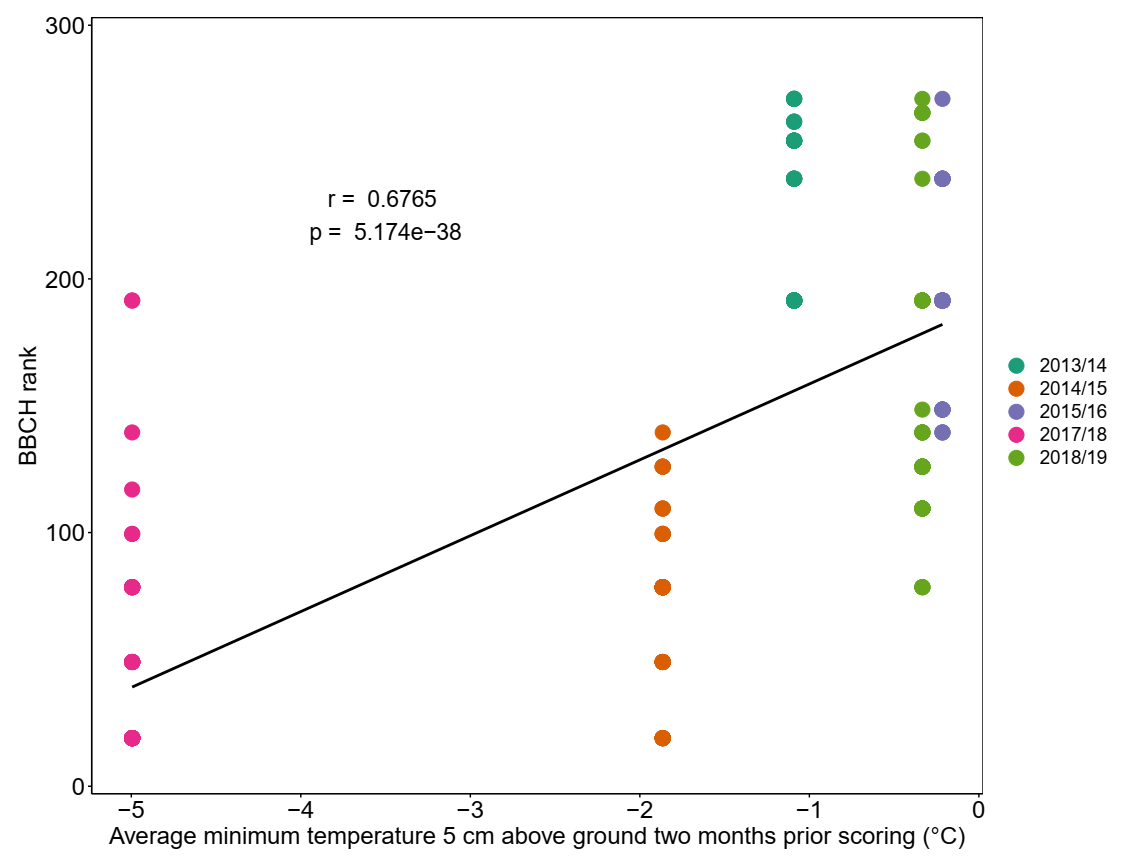


**Suppl. Fig. S3** Correlation analysis of the effect of temperature on the developmental stage of plants in 11 *Arabidopsis thaliana* accessions under field conditions. Ranked BBCH Index was calculated for 2013/14 (turquoise), 2014/15 (orange), 2015/16 (violet), 2017/18 (pink) and 2018/19 (green) for all accessions and plotted against the average temperature at 5 cm above ground in the two months prior to scoring of the plants in each respective year. Data points represent five replicates for each accession and year (n = 274). Spearman correlation coefficient (ϱ) and *P*-value are indicated. Accessions with description as well as their respective freezing tolerance (LT_50_ ACC) are listed in Suppl. Table S1.


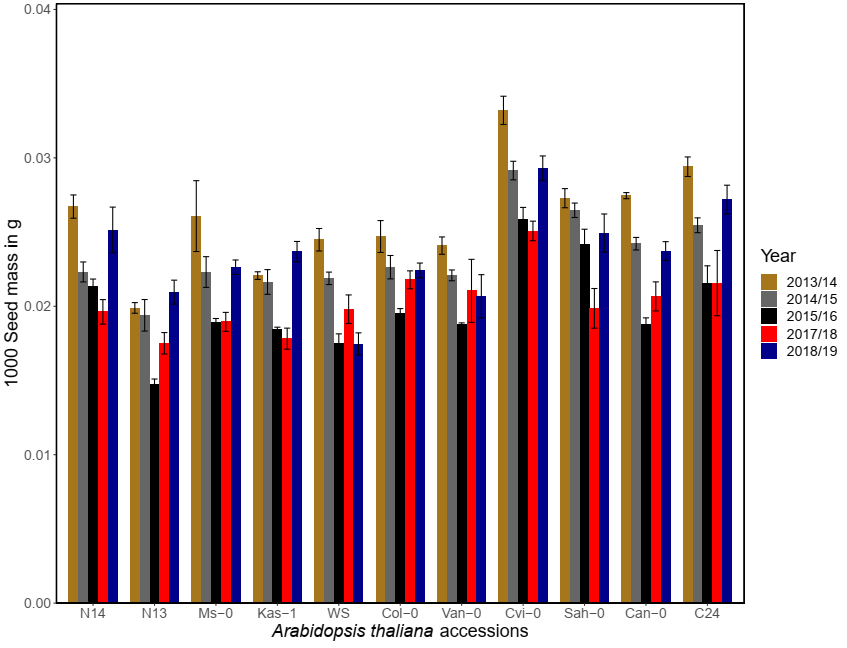


**Suppl. Fig. S4** 1000 Seed mass in 11 *A. thaliana* accessions with different freezing tolerance grown under field conditions in four trials. Accessions are ordered from the most tolerant (N14) to the most sensitive to freezing (C24). Bars represent the average of five biological replicates ± SE.

**
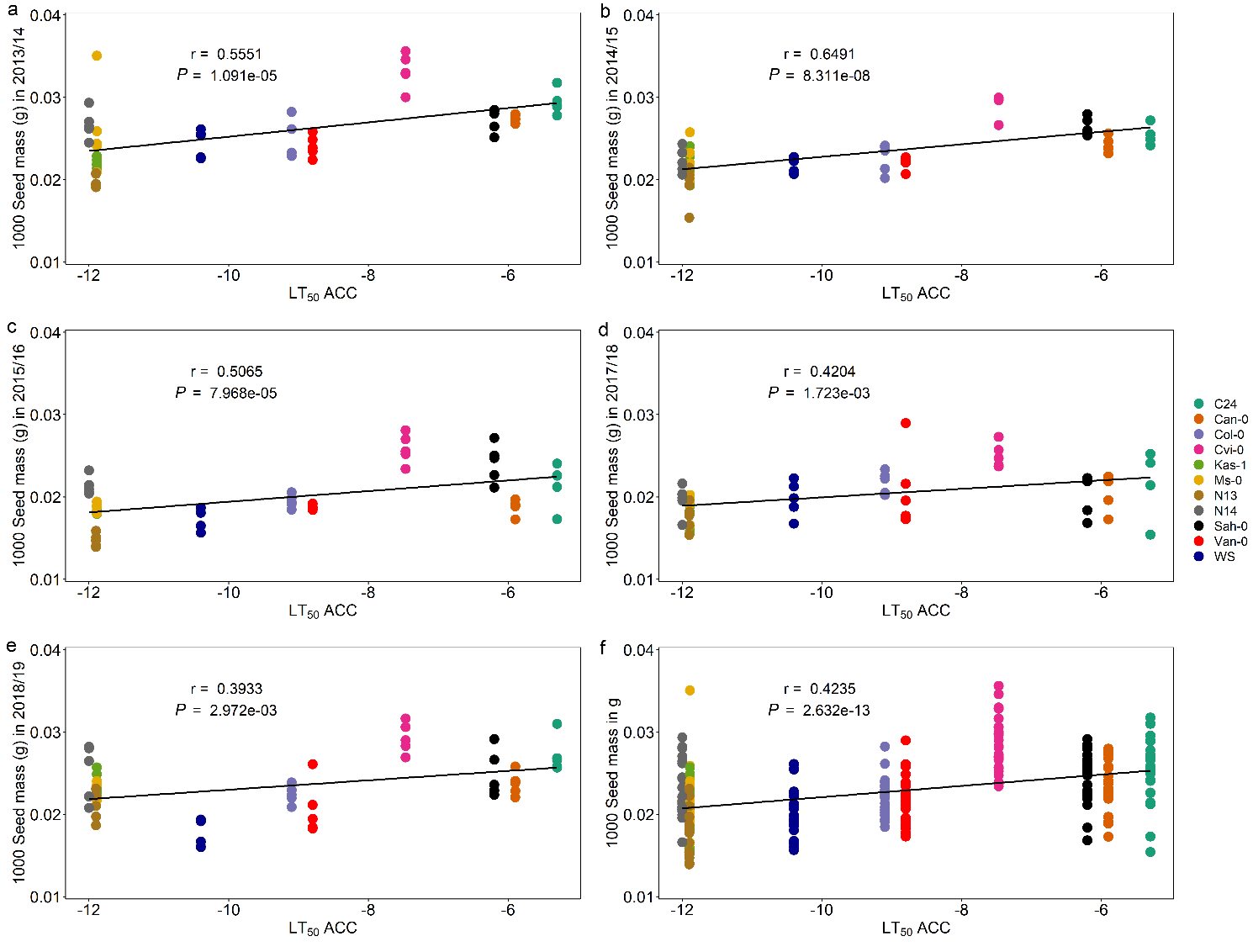
**

**Suppl. Fig. S5** Correlation analysis of cold acclimated freezing tolerance (LT_50_ ACC) with the 1000 seed mass of 11 *A. thaliana* accessions grown under field conditions. 1000 seed mass was determined in the five field trials (**a**: 2013/14, **b**: 2014/15, **c**: 2015/16, **d**: 2017/18, **e**: 2018/19, **f**: All five years combined).Data points represent five replicates for each accession (differently coloured) and year (n = 55). Pearson correlation coefficients (r) and p values are indicated. Accessions with description as well as their respective freezing tolerance (LT_50_ ACC) are listed in Suppl. Table S1.

**
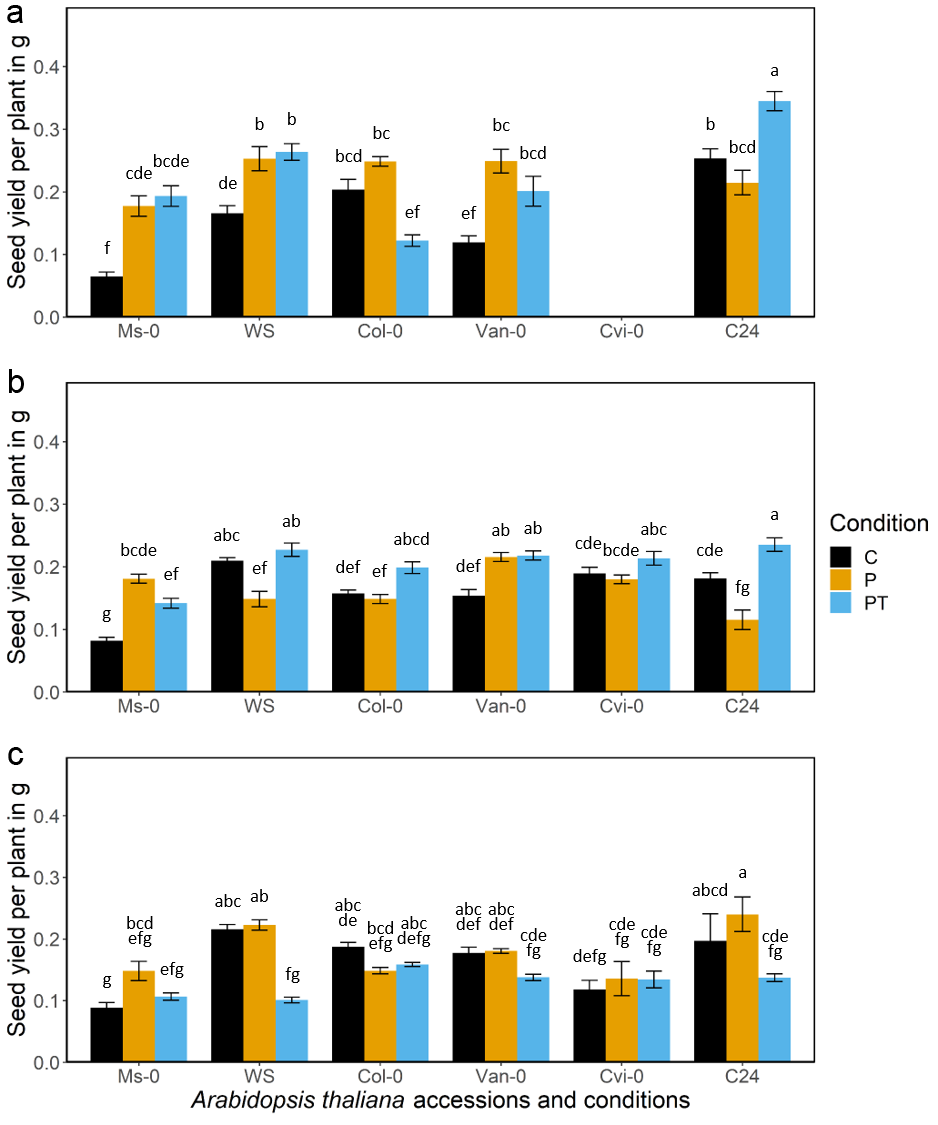
**

**Suppl. Fig. S6** Seed yield per plant for five (2015) or six (2018, 2019) *A. thaliana* accessions with different freezing tolerance grown in the greenhouse under three different conditions. (**a**) Greenhouse Experiment 2015, (**b**) Greenhouse Experiment 2018, (**c**) Greenhouse Experiment 2019. C, control group grown under long day conditions, black bars; P, group that was exposed to cold stress (4°C) for two weeks (priming), orange bars; PT, group that was exposed to cold stress (4°C) for two weeks twice, with an interval of one week of control conditions in between both treatments (priming and triggering), blue bars. Statistically significant differences (ANOVA *P* < 0.05, Tukey HSD *P* < 0.05) were labelled with different letters. Accessions are ordered from the most freezing tolerant (Ms-0) to the most freezing sensitive (C24) to freezing.

**
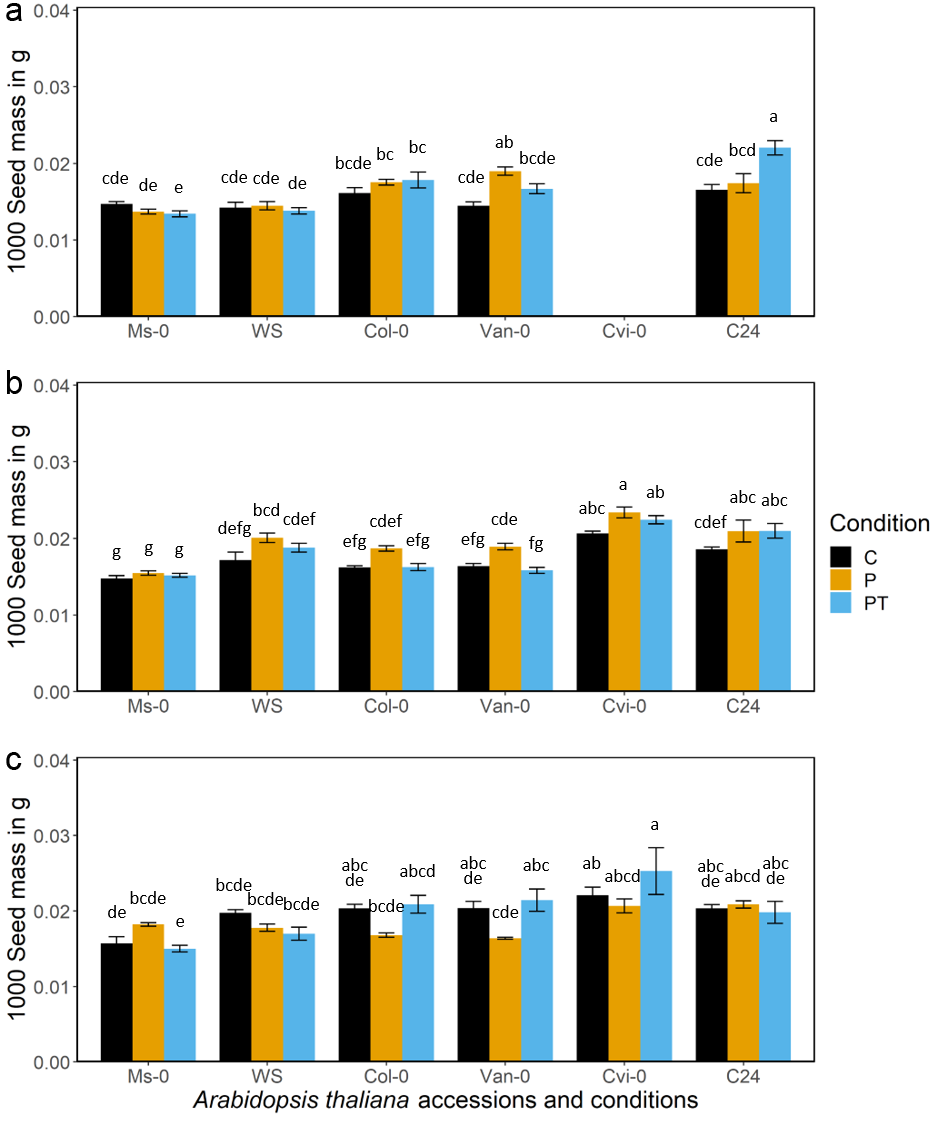
**

**Suppl. Fig. S7** 1000 Seed mass for five (2015) or six (2018, 2019) *A. thaliana* accessions with different freezing tolerance grown in the greenhouse under three different conditions. (**a**) Greenhouse Experiment 2015, (**b**) Greenhouse Experiment 2018, (**c**) Greenhouse Experiment 2019. C, control group grown under long day conditions, black bars; P, group that was exposed to cold stress (4°C) for two weeks (priming), orange bars; PT, group that was exposed to cold stress (4°C) for two weeks twice, with an interval of one week of control conditions in between both treatments (priming and triggering), blue bars. Statistically significant differences (ANOVA *P* < 0.05, Tukey HSD *P* < 0.05) were labelled with different letters. Accessions are ordered from the most freezing tolerant (Ms-0) to the most freezing sensitive (C24) to freezing.
