# Supplementary material for: Cold stress and freezing tolerance negatively affect the fitness of Arabidopsis thaliana accessions under field and controlled conditions
Source: Planta. 2022 Jan 15;255(2):39. doi: 10.1007/s00425-021-03809-8 (PMC8761124; doi:10.1007/s00425-021-03809-8)
Supplement: Supplementary file 2 — Supplementary file2 (DOCX 29 KB) [file 425_2021_3809_MOESM2_ESM.docx]

# **Cold stress and freezing tolerance negatively affect fitness of *Arabidopsis thaliana* accessions under field and controlled conditions**

# Maximilian Boinot^1^, Esra Karakas^1^, Karin Koehl^1^, Majken Pagter^1,2^, Ellen Zuther^1^

# ^1^Max Planck Institute of Molecular Plant Physiology, Am Muehlenberg 1, 14476 Potsdam, Germany

^2^Department of Chemistry and Bioscience, Aalborg University, 9220 Aalborg East, Denmark

*****Correspondence: [zuther@mpimp-golm.mpg.de](mailto:zuther@mpimp-golm.mpg.de)

Planta

**Suppl. Table S1** *A. thaliana* accessions used in the field trials and additionally in controlled greenhouse (in bold) experiments, sorted from highest to lowest acclimated freezing tolerance (LT_50_ACC) (Zuther et al. 2012, 2015). LT_50_, Leakage temperature when 50% damage occurs; NA, non-acclimated conditions; ACC, cold acclimated conditions for 14 days at 4°C).

| Accession | Country of Origin | LT_50_NA | LT_50_ACC | Freezing tolerance |
| --- | --- | --- | --- | --- |
| N14 | Russia | -7.4 | -12.0 | High |
| N13 | Russia | -5.5 | -11.9 | High |
| Ms-0 | Russia | -7.7 | -11.9 | High |
| Kas-1 | Indonesia | -4.7 | -11.9 | High |
| Ws | Russia | -6.0 | -10.4 | High/Intermediate |
| Col-0 | Poland | -5.3 | -9.1 | Intermediate |
| Van-0 | Canada | -6.0 | -8.8 | Intermediate |
| Cvi-0 | Cabo Verde | -5.0 | -7.5 | Low |
| Sah-0 | Spain | -4.7 | -6.2 | Low |
| Can-0 | Spain | -4.3 | -5.9 | Low |
| C24 | Portugal | -4.6 | -5.3 | Low |

**Suppl. Table S2** Germination rates of seed from the 2017 seed batch for all 11 accession. Accessions are ordered alphabetically.

| **Accession** | **Average germination rate in %** | **SE** |
| --- | --- | --- |
| C24 | 99.42 | 0.577 |
| Can-0 | 99.41 | 0.592 |
| Col-0 | 99.40 | 0.600 |
| Cvi-0 | 97.42 | 0.668 |
| Kas-1 | 99.40 | 0.600 |
| Ms-0 | 99.80 | 0.198 |
| N13 | 99.81 | 0.190 |
| N14 | 92.03 | 2.626 |
| Sah-0 | 98.41 | 1.028 |
| Van-0 | 76.27 | 2.696 |
| WS | 65.02 | 4.417 |

**Suppl. Table S3** Schedule for the five field experiments.

| **Year** | **Sowing Date** | **Transfer to Field** | **BBCH Scoring** | **Bagging** |
| --- | --- | --- | --- | --- |
| 2013/14 | 25.10.2013 | 07.11.2013 | 04.04.2014 | 12.05.2014 |
| 2014/15 | 24.10.2014 | 07.11.2014 | 07.04.2015 | 20.-25.05.2015 |
| 2015/16 | 22.10.2015 | 05.11.2015 | 07.04.2016 | 12.05.2016 |
| 2017/18 | 05.10.2017 | 19.10.2017 | 15.03.2018 | 03.-09.05.2018 |
| 2018/19 | 17.10.2018 | 31.10.2018 | 28.03.2019 | 02.-06.05.2019 |
